# Comprehensive Analysis of the Chitinase Family Genes in Tomato (*Solanum lycopersicum*)

**DOI:** 10.3390/plants8030052

**Published:** 2019-02-28

**Authors:** Jun Cao, Xiaona Tan

**Affiliations:** Institute of Life Sciences, Jiangsu University, Xuefu Road 301, Zhenjiang 212013, China; tanxn333@163.com

**Keywords:** chitinase, phylogenetic analyses, evolution, selective pressure, expression, functional network

## Abstract

Chitinase catalyzes the hydrolysis of chitin β-1,4 linkages. However, plants cannot produce chitin, suggesting that plant chitinases do not have the same function as animals. This study investigated the *chitinase* gene family in tomato and divided into eight groups via phylogenetic analyses with Arabidopsis and rice members. Conserved gene structures and motif arrangements indicated their functional relevance with each group. These genes were nonrandomly distributed across the tomato chromosomes, and tandem duplication contributed to the expansion of this gene family. Synteny analysis also established orthology relationships and functional linkages between Arabidopsis and tomato *chitinase* genes. Several positive selection sites were identified, which may contribute to the functional divergence of the protein family in evolution. In addition, differential expression profiles of the tomato *chitinase* genes were also investigated at some developmental stages, or under different biotic and abiotic stresses. Finally, functional network analysis found 124 physical or functional interactions, implying the diversity of physiological functions of the family proteins. These results provide a foundation for the exploration of the *chitinase* genes in plants and will offer some insights for further functional studies.

## 1. Introduction

Chitin is a type of glycopolymer of *N*-acetyl-d-glucosamine in nature, which is widely distributed among the insect exoskeleton, the crustacean shell, and the fungal cell wall [1]. As a molecular model for identifying potential pathogens, chitin participates in the innate immune system of animals and plants. Chitin oligosaccharides have been shown to induce a variety of defensive responses through some chitin elicitor-binding proteins in plants [2,3,4]. Hydrolysis of the chitin was catalyzed by chitinase. Plants do not produce chitin, therefore, plant chitinases have been indicated to hydrolyze the cell walls of pathogens and to release elicitors for defense reactions [5]. Structurally, the chitinase protein adopts a (β/α)_8_ fold with an α-helix and a five stranded β-sheet insertion [6]. Based on their amino acid sequence similarity, plant chitinases are generally divided into six groups [7]. Among these groups, Classes III and V belong to the GH (glycosyl hydrolase) 18 family, and the remains belong to the GH19 family. Class I chitinases have the chitin binding domain (CBD) at the N-terminal region, while Classes II and III do not contain it [8]. Class V possesses two CBDs and a C-terminal extension. Only one half of the CBD was found in Class VI chitinases [9]. The divergent sequences and structures among different groups implied that they may originate from different ancestral genes [10,11].

Chitinase activity can be induced by infection of the tissue with fungal or pathogens in many plants [12,13,14,15,16]. Through blocking mycelium cells and inducting downstream defense pathways, the induced plant chitinases are involved in the defense response against pathogen and fungal infection [17]. Overexpression of the *chitinase* and the *glucanase* genes can enhance the protection against fungal attack in transgenic tobacco [18]. Moreover, chitinases are also involved in the growth and development of plants. Mutation of the Arabidopsis Class II *chitinase* causes cell morphological abnormalities and length changes of the internode and root [19]. Similar study on chitinase affecting root growth has also been reported in rice [20]. Mutation of the *OsCTL1* gene reduces cellulose content and mechanical strength, suggesting a possible mechanism for cellulose biosynthesis and cell wall remodeling [21]. Other biological processes, such as cell separation or loosening, embryonal development, and programmed cell death, are associated with plant chitinase activity [22,23,24,25,26]. In addition, some plant hormones and abiotic stress also regulate the expression and activity of chitinases [27,28,29]. For instances, jasmonates and wounding can induce the expression of *BjCHI* and *SafChia* genes [30,31]. Some *chitinases* are upregulated by heavy metals including cadmium, and arsenic [32]. Moreover, salt, cold and drought stresses promote the transcription of some *chitinase* genes [28,31]. All these suggest that chitinases may be involved in a variety of growth processes and stress responses.

Plants are easily attacked by various pathogens and other environmental factors, and it seems reasonable to expect that selection will favor functional divergence in the evolution process. A previous study indicated that positive selection can drive the divergence of *ChiA* and *ChiB* duplicated genes in the GH19 family [33]. This variation has spurred plants to obtain some ability to recognize a wide diversity of pathogens [34]. The rapid evolution of plant *chitinases* implies the coevolutionary interactions between plants and pathogens. Furthermore, the changes of these proteins can explain the diversity of disease resistance [35]. In the evolutionary process, several chitinases have retained the chitin-binding ability while lost hydrolyzing ability, which may affect a number of biological processes as described above [11].

As one of the most economically important vegetables in the world, tomato (*Solanum lycopersicum*) has been chosen as one model species for studies of fruit development [36], domestication [37], and stress responses [38]. A total of 25 and 49 *chitinase* family genes have been identified in Arabidopsis and rice, respectively [29]. Only few tomato *chitinases* have been functionally identified to date [23,39] and no systematic investigation of chitinase protein family has been reported in the tomato. The completed sequencing of the tomato provides an opportunity to deduce the *chitinase* gene family and infer its evolutionary history [40]. This study identified 43 tomato *chitinases* via database analysis. Their phylogeny, structure, chromosomal location and duplication, and synteny were then investigated, followed by selective pressure analysis and expression profiles. Finally, a functional network analysis of the tomato chitinase proteins was also conducted. This study provides several useful insights for further evolutional and functional investigation of this gene family. 

## 2. Materials and Methods

### 2.1. Identification of the Chitinase Genes in Tomato

To identify potential *chitinase* genes in the tomato plant genome (*S. lycopersicum*), first, all chitinase sequences of Arabidopsis and rice [29] were used as queries to perform BLAST searches against the tomato database [40] in the phytozome database (http://www.phytozome.net) [41] with a threshold of -1 E. Furthermore, all candidate proteins were further used to perform new queries in this database. SMART [42] and Pfam [43] were used to further examine the obtained sequences, and those without common domains (PF00182, PF00187, or PF00704) were excluded. The physicochemical parameters and subcellular localization of the chitinase proteins were predicted using the ProtParam tool [44] and the TargetP 1.1 server [45], respectively.

### 2.2. Phylogeny, Gene Organization, and Conserved Motif Analysis of the Chitinase Gene Family

Multiple sequence alignment of all predicted chitinase proteins was performed with the MUSCLE method [46]. Next, MEGA6 [47] was used to construct a neighbor joining (NJ) tree with 1000 bootstrap replications, a *p*-distance substitution model, and pairwise deletion gaps parameters. Gene organization was inferred from the phytozome database (http://www.phytozome.net) by comparing the genomic sequence and the CDS sequence of *chitinase* members. Additionally, the MEME program [48] was used to identify the conserved motifs with the following parameters: zero or one motif in each sequence, six and 50 width of motifs, and a maximum of 12 motifs.

### 2.3. Chromosomal Location and Duplication Time Inference

Annotation information of the *chitinase* genes in the tomato database was used to determine their chromosomal locations. *Chitinase* paralogous gene pairs were determined via their phylogeny and were used as reference for pairwise DNA coding sequence alignments using the MUSCLE method [46]. K-Estimator 6.0 [49] was used to estimate *K_a_* (the number of nonsynonymous substitutions per nonsynonymous site) and *K_s_* (the number of synonymous substitutions per synonymous site) values for these pairwise alignments. Finally, the *K_s_* value was used to calculate the approximate date of the duplication event (T = *K_s_*/2λ), assuming clock-like rates (λ) of 1.5 × 10^−8^ synonymous/substitution site/year for tomato [50].

### 2.4. Analyses of Synteny

The SynMap server of the CoGe [51] was used to identify syntenic gene blocks between Arabidopsis and tomato with the following parameters: DAGchainer algorithm, a maximum distance of 20 genes between two matches, and a minimum of five aligned gene pairs.

### 2.5. Site-Specific Selection Assessment and Testing

The selective pressure was estimated by calculating the synonymous rate (*K_s_*) and the nonsynonymous rate (*K_a_*) at each codon. Three evolutionary models [M8 (beta + w ≥ 1), M7 (beta), and M5 (gamma)] were used to describe how the characteristics evolve in probabilistic terms using a Bayesian inference approach [52]. Each model used different biological assumptions to assume a statistical distribution that accounts for heterogeneous *K_a_/K_s_* values among sites. Next, eight discrete categories were used to approximate the distribution. Finally, the *K_a_/K_s_* values were computed by calculating the expectation of a posterior distribution [52]. The Phyre2 Server [53] was used to predict the three-dimensional structure of the chitinase protein (Solyc10g055820.1) in the GH19-A group. I-Mutant2.0 [54] was then used to estimate the effects of point mutations on protein folding stability. The free energy change value [∆∆G (kcal/mol)] was used to infer the protein stability. When ∆∆G exceeds zero, the stability of the mutant protein increases; in contrast, when ∆∆G is below zero, the stability decreases.

### 2.6. Transcriptome-Based Expression Analysis

Transcriptome data (GSE33507) was used to investigate the expression analysis of the tomato *chitinase* genes in different development stages. Two to four biological replicates were performed. The average fragments per kilobase of transcripts per million fragments mapped (FRKM) was used as the unit of measurement to estimate the expression levels of each transcript. The Log2 ratio of the sample signal to control signal was used to indicate its relative expression level. The HeatMapper Plus tool of BAR (http://bar.utoronto.ca) was used to access their relative expression levels.

### 2.7. Plant Materials, Stress Treatments, RNA Isolation, Quantitative Real-Time PCR (qRT-PCR) Analysis, and Promoter Sequence Analysis

One-week-old tomato (*S. lycopersicum* L. cv Hezuo 908) seedlings were used to examine the expression patterns of *chitinase* genes under different stress treatments. Plants were grown in liquid MS culture in a plant growth chamber at 23 ± 1 °C with a 14-h light/10-h dark photoperiod. Tomato seedlings were kept at 4 ± 1 and 42 ± 1 °C for 3 h for cold and heat treatments, respectively. Tomato seedlings were put in 150mM NaCl for 24 h for salt stress. For the drought treatment, the seedlings were dried between folds of tissue paper at 23 ± 1 °C for 3 h. Control (CK) seedlings were grown at 23 ± 1 °C with normal irrigation. For the biotic treatment, we first cultured the mycelia of *Sclerotinia sclerotiorum* on PDA medium for about four days. The mycelia of ~3 mm agar plugs in diameter were excised from the edges of growing colonies and were upended onto the tomato leaves for 24 h and 48 h for pathogen infection treatment, respectively. We used the normally grown tomato leaves as controls. Three replicates were performed per sample. After different abiotic and biotic stress treatments, total RNA of each sample was extracted with the Trizol total RNA extraction kit (Sangon, Shanghai, China, SK1321) and treated with DNase I (TakaRa, Dalian, China). Reverse transcription was performed using M-MLV (TakaRa) with random primers. Quantitative assays were performed on each cDNA dilution using the SYBR Green Master Mix (TakaRa) with an ABI 7500 sequence detection system, according to the manufacturer’s protocol. Eleven tomato *chitinase* genes were selected for qRT-PCR analysis with the expression level of the *Actin* gene (*Solyc01g104770.2*) as endogenous control. The specific primers are shown in Table S1. The 2^−∆∆CT^ method [55] was used to calculate the relative expression level of each sample. Through comparing the expressed sequence tag with the genomic sequence, we first define the TSS (transcription start site) of each *chitinase* gene in tomato, Arabidopsis, and rice. Here, 1000 bp upstream promoter sequences were used to identify their *cis*-elements using PLACE [56].

### 2.8. Network Assembly

The STRING database (http://string-db.org) [57] was used to assemble the protein–protein interaction networks. This database includes several interaction sources, such as, textmining, experiments, coexpression, neighborhood, fusion, co-occurrence databases, and so on [57]. All predicted tomato chitinase proteins were submitted to this database. The minimum required interaction score was set to medium confidence (0.400). Max number of interactors showed no more than 20 on the first shell, and no more than 10 on the second shell.

## 3. Results

### 3.1. Identification and Phylogenetic Analysis of the Chitinase Gene Family in Tomato

To identify the putative *chitinase* family genes in the tomato genome, a BLAST search of the phytozome database (http://www.phytozome.net) was first performed using the with methods described above. As a result, 43 putative *chitinase* genes were identified in tomato. Most of these tomato *chitinase* genes encode highly hydrophilous residues of 60–386 amino acids in length, with a predicted isoelectric point (pI) range of 4.41 to 9.6 (Table 1). Further subcellular localization prediction indicated that more than 79% of the candidate chitinase proteins are likely localized in the secretory pathway, which is similar to the results of Arabidopsis and rice chitinases [29].

**Figure 1:**
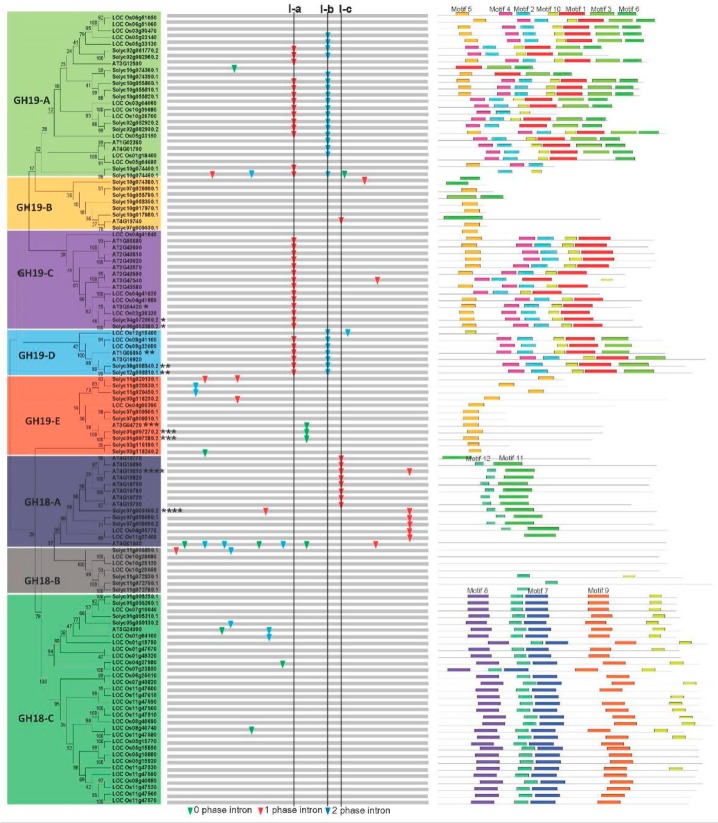
Phylogenetic relationship, gene structure, and motif composition of the *chitinase* genes. The phylogenetic tree is constructed and classified into eight groups. Classification of GH18 and GH19 is based on the SMART and Pfam databases [42,43]. Four syntenic paralogs were marked with * for pairs 1, ** for pairs 2, *** for pairs 3, and **** for pairs 4 as indicated in Figure 3. The insertion positions of 0, 1, and 2 phase introns are marked with green, red, and blue inverted triangles, respectively. Different motifs predicted by MEME [48] of the chitinase proteins are displayed by different colored boxes.

To assess the evolutionary relationship of the *chitinase* genes in the tomato, Arabidopsis, and rice, phylogenetic analyses were performed based on a NJ method using MEGA6 [47]. The 120 predicted chitinase proteins were classified into two major evolutionary branches: GH19 and GH18, which were further divided into the following eight groups: GH19-A, GH19-B, GH19-C, GH19-D, GH19-E, GH18-A, GH18-B, and GH18-C (Figure 1). Further evidences, such as gene organization and conserved motif distribution (described below), also support this classification. The GH18-C group is the largest with 32 members, representing 26.7% of the total number of *chitinase* genes, while GH19-D and GH18-B groups are the smallest, with only seven genes. In addition, one eudicot-specific *chitinase* clade formed the GH19-B group (Figure 1). The distribution of the *chitinase* genes in different species and groups was also assessed (Table S2). Similar to Arabidopsis and rice, tomato *chitinases* are not distributed evenly in these eight phylogeny groups. Nearly half of the tomato *chitinase* genes are distributed in GH19-A and GH19-E groups. With regard to rice, ~53% of the chitinase members are distributed in GH18-C.

### 3.2. Gene Organization and Conserved Motif Distribution of the Chitinases

To further examine the organizational diversity of these *chitinase* genes, their exon–intron structures were inferred by comparing both their coding sequences and their genome sequences. A detailed illustration of the *chitinase* gene organization is shown in Figure 1. In general, the *chitinase* gene organization was well conserved, especially in CH19-A and CH19-D groups, which supports a common origin. Furthermore, three introns (I-a, I-b, and I-c) were designated to demonstrate the feature of exon–intron evolution of chitinases. Intron I-a is conserved among most members of the putative *chitinase* genes in the groups GH19-A, GH19-B, and GH19-D. Intron I-b was only found in the groups GH19-A and GH19-D. Intron I-c is specific for Group CH18-A. In addition, some intron loss or gain has occurred within several evolutionary branches (Figure 1).

To further examine the diversification of chitinase proteins, several conserved motifs were investigated using MEME [48]. As a result, 12 conserved motifs were found in the predicted chitinase proteins (Figure 1; Table S3). Similar motif compositions of each group not only provided additional evidence in support of the results of the phylogenetic analyses, but also implied functional relevance. Furthermore, several distinct motifs were found in specific groups. For instance, motifs 7, 8, and 9 are restricted to the CH18-C group, and motif 11 is unique to the GH18-A group. Interestingly, we did not find any of these 12 motifs in most members of GH18-B group, suggesting a high degree of divergence between them.

### 3.3. Chromosomal Localization, Gene Duplication and Synteny Analyses

**Figure 2:**
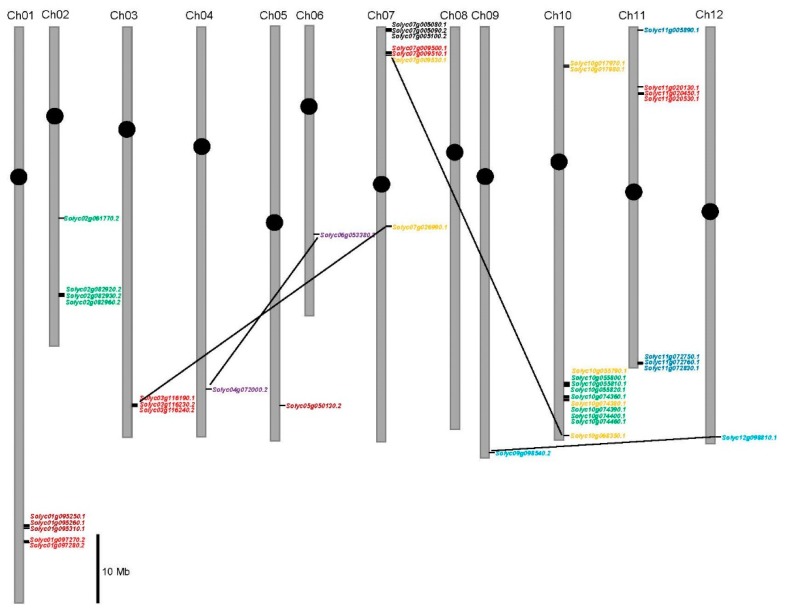
Positions of the *chitinase* family genes on the tomato chromosomes. Scale stands for a 10 Mb chromosomal distance. Segmental duplicate genes are linked by a black line. *Chitinase* members of different groups are marked with different colors: GH19-A members in green; GH19-B in saffron yellow; GH19-C in purple; GH19-D in light blue; GH19-E in red; GH18-A in black; GH18-B in dark blue; and GH18-C in deep red, respectively.

To further investigate the relationship between the genetic divergence and gene duplication of the tomato *chitinase* gene family, their chromosomal locations were determined. The results indicated that 43 tomato *chitinase* genes are located on 11 different chromosomes, and chromosome 8 does not contain any *chitinase* gene (Figure 2). Among the identified *chitinase* genes, twelve are present on chromosome 10; seven on chromosomes 7 and 11; five on chromosome 1; four on chromosome 2; three on chromosome 3; and one on each of chromosomes 4, 5, 6, 9, and 12 (Figure 2). Furthermore, several *chitinase* gene clusters containing 34 tandem members were found on chromosomes 1, 2, 3, 7, 10, and 11. Consequently, over 79% of tomato *chitinase* genes originate from tandem duplication. Four pairs of paralogous *chitinase* genes (*Solyc03g116190.1*-*Solyc07g026990.1*, *Solyc04g072000.2*-*Solyc06g053380.2*, *Solyc07g009530.1*-*Solyc10g068350.1*, and *Solyc09g098540.2*-*Solyc12g098810.1*) were putative segmental duplication events (Figure 2).

To better understand the evolutionary history of the *chitinase* family, the *K_s_* values were used to estimate the duplication timing of 16 pairs of paralogous genes (Table 2). The *K_s_* values of the tomato *chitinase* paralogues ranged from 0.01677 to 1.45233, suggesting that these duplication events occurred approximately 0.56–48.41 million years ago (Mya). Furthermore, duplication events were found in eight of 16 pairs of paralogous *chitinase* genes to have occurred at approximately 11.91–27.94 Mya. This period coincided with the recent large-scale duplication event of the tomato genome [50], implying that the expansion of the tomato *chitinase* gene family mainly occurred during the recent large-scale duplication event period. The initial (*Solyc07g005080.1*–*Solyc07g005090.2*) and the final (*Solyc10g017970.1*–*Solyc07g017980.1*) tandem duplication occurred at about 48.41 and 0.56 Mya on the *chitinase* cluster region of the tomato chromosomes 7 and 10, respectively.

**Figure 3:**
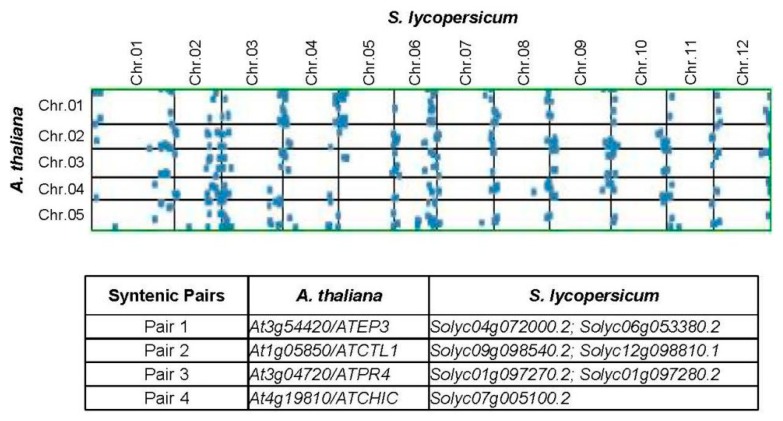
Synteny analyses of the *chitinase* genes in Arabidopsis and tomato genomes. Four syntenic paralogs were found and also marked with * for pairs 1, ** for pairs 2, *** for pairs 3, and **** for pairs 4 in Figure 1.

Identification of potential orthologue genes contributes to the preliminary understanding of the chitinases in other species. Next, synteny analyses were performed between Arabidopsis and tomato genomes using the SynMap server [51]. Four ortholog pairs were identified in syntenic blocks (Figure 3). *Solyc04g072000.2* is microsyntenic to *Solyc06g053380.2* and syntenic to *At3g54420* (*ATEP3*) (ortholog pair 1). *At1g05850* (*ATCTL1*) is orthologous to *Solyc09g098540.2* and *Solyc12g098810.1* (ortholog pair 2). *ATCTL1* is involved in both lignin accumulation and root system architecture [58,59]. *Solyc09g098540.2* and *Solyc12g098810.1* belong to GH19-D and are likely connected to the development of plant root cell wall, since *At3g16920* (*ATCTL2*) is clustered in this clade [60]. In addition, both *Solyc01g097270.2* and *Solyc097280.2* are syntenic to *At3g04720* (*ATPR4*), which controls the resistance to pathogen infection [61].

### 3.4. Selective Pressure Analysis among Different Amino Acid Sites

**Figure 4:**
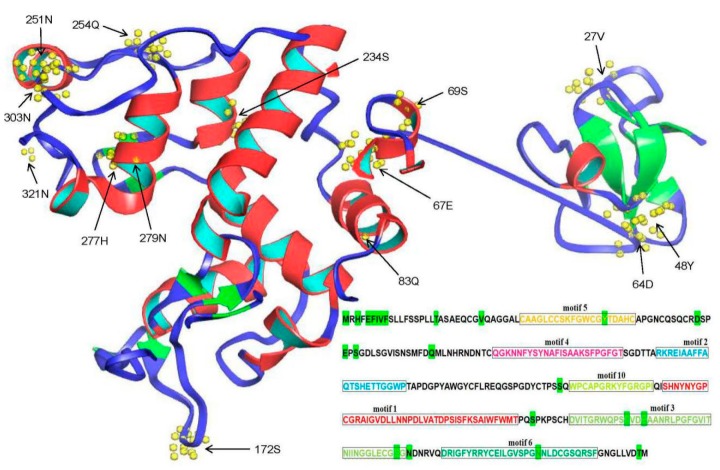
Distribution of positive selection sites of chitinase members in CH19-A group predicted by M5 model. Solyc10g055820.1 was used to predict the tertiary structure of the chitinase protein with the Phyre2 Server [53]. Predicted positive selection amino acid sites are displayed and marked with arrowheads or bright green. In addition, distribution of the seven conserved motifs are also marked different colors here.

During the process of evolution, positive selection sites often affect the protein structure and their function [62]. Phylogenetic results indicated that eight groups were generated after *chitinase* duplication in the three investigated plant species. To further explore which amino acid change was subjected to positive selection after duplication, variable *K_a_*/*K_s_* values were investigated among different chitinase sites in each group. The results indicated that the *K_a_/K_s_* values differed for each group (Table 3). Compared to the values for the others, the *K_a_/K_s_* values are comparatively higher in GH19-B, GH19-E, and GH18-A, indicating a faster evolutionary rate within the three groups (Table 3). Despite these differences, all *K_a_/K_s_* values are below 1, suggesting that the chitinase proteins are under purifying selection. However, some positive selection sites, such as chitinase members in GH19-A and GH19-B (as predicted by the M5 model) and GH19-E, GH18-A, and GH18-C (as predicted by the M5 and M8 models), were also found in this analysis (Table 3). However, no positively selected sites were found with the M7 selection model. As an example, a detailed distribution position of the predicted positive selection sites in GH19-A via the M5 model is shown in Figure 4. Twenty-two sites were found to be under positive selection. Among them, eight sites were located in the signal peptide region of the chitinase protein (Solyc10g055820.1). Since the signal peptides will be removed in the mature protein, the tertiary structure of this chitinase does not contain this segment. Therefore, only 14 predicted amino acid sites are shown here (Figure 4). Further analyses indicated that six sites were located in α-helixes and five in β sheets. As described above, seven conserved motifs (motifs 5, 4, 2, 10, 1, 3, and 6) were found in most members of the GH19-A group (Figure 1). Of the 22 predicted positive selection sites, only five sites were located in the conserved motifs, containing three sites (251N, 254Q, and 277H) in motif 3 and one site (48Y and 303N) in motifs 5 and 6, respectively. In summary, more than 77% of the positive selection sites are located in the less conserved region of the chitinase protein. To further investigate whether these positive selection sites can cause changes in protein stability, I-Mutant2.0 [54] was used to estimate the effects of point mutations on the protein folding stability on the chitinase protein (Solyc10g055820.1). As shown in Table S4, most of the detected sites under positive selection can decrease the stability of the chitinase protein.

### 3.5. Divergent Expression Profile of Tomato Chitinase Genes in Different Tissues

**Figure 5:**
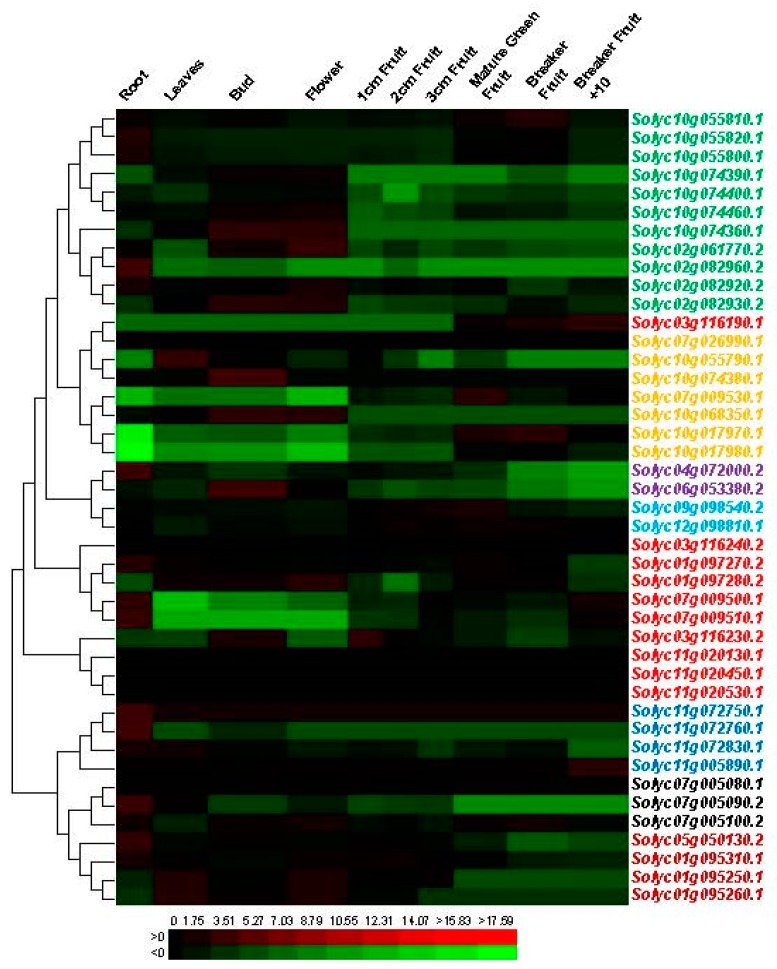
Expression patterns of the *chitinase* genes in different developmental stages of tomato. The Log2 ratio of the sample signal to control signal was used to indicate its relative expression level. Heat maps reflect the strength of relative expression. Members of different groups are marked with different colors (please refer to Figure 2).

To further understand the roles of specific *chitinase* genes in different tomato tissues, we investigated the expression profiles of these genes using available transcriptome data (GSE33507). Transcription profiles of the *chitinase* genes were collected and analyzed in 10 different tissues (Figure 5; Table S5). The results showed that the *chitinase* genes showed diverse expression profiles among the different tissues, suggesting distinct roles for a variety of developmental stages. Several *chitinase* genes were significantly abundant in some tissues. For instance, *Solyc10g074360.1*, *Solyc02g082930.2*, *Solyc10g068350.1*, and *Solyc06g053380.2* transcripts accumulated more during the bud and flower stages than in the other tissues. The highest level of *Solyc02g082960.2*, *Solyc04g072000.2*, *Solyc11g072760.1* and *Solyc07g005090.2* gene transcripts are found in the root, whereas expression of the *Solyc02g082930.2*, *Solyc11g005890.1*, and *Solyc07g009530.1* was highest at the fruit development stage. As described above, 16 pairs of paralogous *chitinase* genes in tomato were identified to originate from duplication (Table 2). We also examined the expression profiles of these paralogous genes and found that none of them showed similar expression patterns (Figure 5).

### 3.6. Divergent Expression Profile of Tomato Chitinase Genes under Various Abiotic and Biotic Stresses

**Figure 6:**
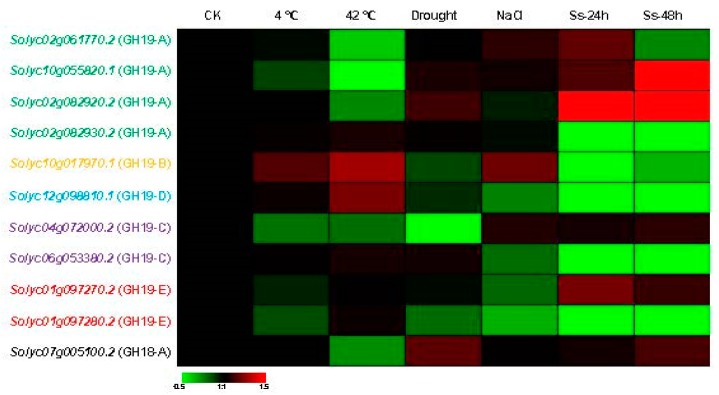
Expression profiles of the eleven tomato *chitinase* genes under different biotic and abiotic stresses by qRT-PCR. The fold change between the treated sample and the control sample as the relative expression level. Heat maps reflect the strength of relative expression. Ss stands for *S. sclerotiorum*. *Chitinase* genes of different groups are marked with different colors (please refer to Figure 2).

To further determine the involvement of tomato *chitinase* genes in the response to various stresses, we further analyzed the expression patterns of 11 genes under low temperature (4 °C), high temperature (42 °C), drought, salt, and *S. sclerotiorum* stresses via qRT-PCR (Figure 6; Table S6). The expression levels of seven and five tested *chitinase* genes were enhanced under low and high temperature stress, respectively. More than half of the analyzed tomato *chitinase* genes showed a higher expression level under drought stress. Under salt treatment, 10 of the 11 selected tomato *chitinase* genes displayed elevated expression, while *Solyc01g097280.2* showed to be downregulation. For *S. sclerotiorum* infection, the expression levels of more than 64 percent detected *chitinase* genes increased during this pathogen stress, and this trend was particularly evident after 48 h of treatment (Figure 6). Interestingly, the *Solyc10g017970.1* gene was upregulated under low temperature, high temperature, salt, and *S. sclerotiorum* stresses, suggesting that this *chitinase* gene may be involved in these biotic and abiotic responses. The *Solyc04g072000.2* gene showed an enhancement of the transcript level under salt and *S. sclerotiorum* stresses and the expression level of this gene decreased in response to the other three stresses, implying that the *Solyc04g072000.2* gene may be involved in the salt and pathogen tolerance of the tomato. In addition, the functional divergence of duplicated genes was also investigated. Two pairs of duplicated *chitinase* genes (*Solyc02g082920.2*-*Solyc02g082930.2* and *Solyc01g097270.2*-*Solyc01g097280.2*) did not show similar expression patterns in response to these stresses. For examples, high temperature and pathogen infection can induce the expression of *Solyc02g082930.2* gene but decrease the transcript level of the *Solyc02g082920.2* gene.

To further explore the potential regulatory mechanism of the *chitinase* genes responsive abiotic stress in tomato, Arabidopsis and rice, we also identified their *cis*-elements using the PLACE [56]. A few abiotic response regulatory elements were found here. They are cold responsive element (S000407), low temperature-responsive element (S000157), heat shock element (S0000030), cold/drought-responsive element (S000153), drought-responsive element (S000414 and S000174), salt- and pathogen-responsive element (S000453) and pathogen-responsive element (S000390). The results indicated that all *chitinase* genes contained multiple cis-elements in their promoters (Table S7), suggesting these abiotic stresses regulated their expression. And we cannot find any same or highly similar distribution of these regulatory elements among paralogs, implying divergent expression patterns in the duplicated genes.

### 3.7. Protein–Protein Interaction Network Analysis of the Chitinases in Tomato

**Figure 7:**
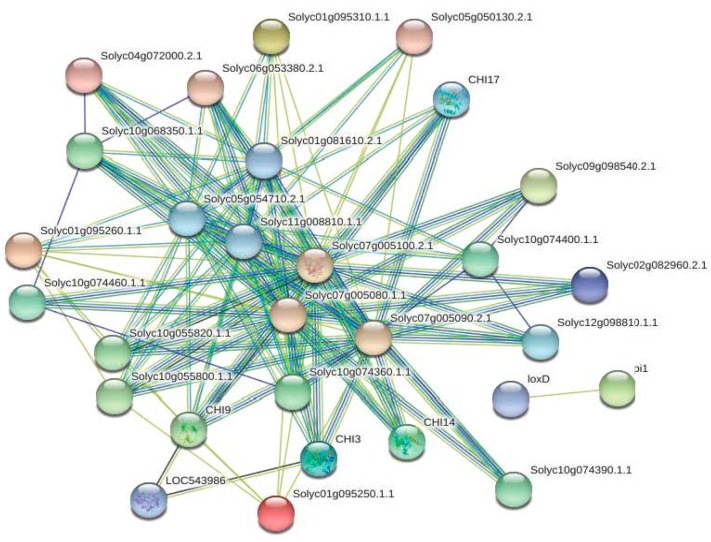
Functional network assembly of the tomato *chitinase* genes. 124 interactions were exhibited among a total of 29 genes.

To further investigate which proteins can potentially interact with members of the chitinase family, a protein interaction network was assembled with the STRING database [57]. The network was based on either experimental or predicted interactions. As a result, 24 of the 43 tomato chitinases appeared in the network, exhibiting 124 interactions by a total of 29 unique genes (Figure 7; Table S8). Among these, 95 interactions occurred between the tomato chitinase proteins and Solyc07g005080.1 and Solyc07g005100.2 interacted with other 20 chitinases (Figure 7; Table S8). Beta-hexosaminidase, lipoxygenase and beta-1,3-glucanase were predicted as the main interaction partners of tomato chitinases. For instances, three beta-hexosaminidases (Solyc01g081610.2, Solyc05g054710.2, and Solyc11g008810.1.1) were predicted to interact with chitinases (CHI3: Solyc02g082920.2; CHI9: Solyc10g055810.1; CHI14: Solyc02g061770.2; CHI17: Solyc02g082930.2). Solyc01g097270.2 (pi1) was predicted to interact with loxD (Solyc03g122340.2) in tomato. Furthermore, one tomato beta-1,3-glucanase protein (LOC543986: Solyc01g008620.2) was observed to interact with both CHI3 (Solyc02g082920.2) and CHI9 (Solyc10g055810.1). These results implied functional diversity of tomato chitinases.

## 4. Discussion

In this study, tomato *chitinase* genes were identified and compared with data in Arabidopsis and rice. Phylogenetic analyses divided them into eight groups. This is different from previous classification of chitinase proteins that were divided six groups (Classes I–VI) according to their sequence similarity [7]. We also aligned the tomato chitinase protein sequences, and found some signatures of GH18 and GH19 family and catalytic or binding residues (Figure S1). By comparing our data with previous classifications [7,63], we found that GH18-A and GH18-C members in this study belong to the Class V and Class III, respectively. Chitinase members in GH19-A and GH19-C have the catalytic residues, they should be classified as the Class I. CH19-D members should belong to the Class II because they do not contain chitin-binding domain. Gene organization and conserved motif distribution provide additional evidences for the evolutionary relationships of multigene families [64,65]. Intron gain and loss can increase the complexity of gene organization throughout evolution [66]. The diversification of the exon–intron organization of *chitinase* family genes among different groups may contribute to the structural diversity of this family during plant evolution. The structural diversity of chitinase proteins (Figure 1; Table S3) can also mediate a number of biological processes such as stress responses, growth, and developmental operations, which has led to neofunctionalization [11]. Moreover, a similar motif composition was found in each group, suggesting functional relevance. A previous study has shown that the DxDxE sequence of motif 11 is important for the catalysis in family GH-18 enzymes [67]. At the same time, this diverse motif composition implies functional diversification among different groups.

Chromosomal localization and gene duplication studies showed that 79% of tomato *chitinase* genes originated from tandem duplication (Figure 2). In other words, tandem duplication predominated in tomato *chitinase* gene expansion during evolution. This is similar to the results of Arabidopsis and rice, where most of the *chitinase* genes were also reported to originate from tandem duplication events [29]. Duplication of chromosomal segments can amplify the number of family genes, while the duplication of family genes (such as tandem duplication) can also affect the genetic composition of the chromosomal segments. Over the course of evolution, the duplicated genes will change their functions, i.e., genetic divergence of the duplication genes will occur throughout evolution [68]. Next, synteny analyses between the Arabidopsis and tomato genomes were also found several potential orthologue genes: *ATEP3*, *ATCTL1*, *ATCTL2*, and *ATPR4* (Figure 3). The transcript of the *ATEP3* gene was accumulated rapidly after inoculation with *Xanthomonas campestris*, suggesting that ATEP3 protein is involved in the initial events of the hypersensitive reaction [17]. However, specific abiotic stresses did not significantly affect *ATEP3* gene expression [28]. Based on this, both of these chitinases in tomato might be responsible for host defense against pathogens, but not for abiotic stress tolerance. *ATCTL1* is involved in both lignin accumulation and root system architecture [58,59]. In addition, *ATCTL2* and *ATPR4* are connected to the development of the plant root cell wall and the resistance to pathogen infection, respectively [60,61]. In this study, both *Solyc09g098540.2* and *Solyc12g098810.1* were found to cluster with *ATCTL2*, and *Solyc01g097270.2* and *Solyc097280.2* are syntenic to *ATPR4.* Therefore, these identified tomato chitinases might have functions in cell wall architecture and disease resistance, which requires further experimental verification.

Positively selected amino acid sites can alter protein structure and function in evolution [50]. Some studies have indicated that purifying selection promotes subfunctionalization of the duplicated genes, and positive selection accelerates neofunctionalization of the duplicated genes [66,68]. In this study, we found that the selective pressure from different *chitinase* groups are different (Table 3), and several positive selection sites located at different protein positions were also identified (Figure 4). It suggests that selection spurs the amino acid diversity at some residues. Interestingly, we also found that most positive selection sites were located to the variable regions of chitinase proteins, implying that these sites might change the protein structures and thus increase their function divergence.

Furthermore, the divergent expression profile of tomato *chitinase* genes was investigated in different tissues and under some biotic and abiotic stresses (Figure 5 and Figure 6), suggesting this gene family may be involved in a variety of physiological processes [69,70]. For duplicated genes, a change of expression pattern is one of the characteristics of the functional divergence. Several studies have reported that different transcription patterns occurred in duplicated genes [66,71]. In our study, the diverse expression profile of the *chitinase* genes might be the result of subfunctionalization or neofunctionalization processes in tomato. Over the process of biological evolution, one copy of these duplicated genes may acquire new functions, while the other may maintain the original function. Alternatively, both copies may undergo subfunctionalization and are uniquely expressed in different tissues or under different stresses [72,73]. Over the process of evolution, the loss or increase of specific domains (such as CBD) may cause functional changes in the family protein. Consequently, the chitinase family protein can not only hydrolyze chitin, but also be involved in the growth and development of plants as described above. The number increase and functional divergence of *chitinase* family genes are an adaptation of organisms to external environmental changes. All of these findings suggest that functional divergence has occurred among these duplicated genes, and that the products of these genes might play different roles in the tomato development or in a specific stress response. In addition, these 11 genes were distributed in six of the eight identified groups with different motif distributions (Figure 1). The structural difference may affect their functions.

Network analysis of the tomato *chitinases* indicated that most interactions occurred between the tomato chitinase proteins (Figure 7; Table S8), indicating that some *chitinase* genes may be neighborhood, co-occurrence, or coexpressed. Such a joint participation characteristic of the tomato *chitinase* genes is of great significance for the defense response against pathogens and for the regulation of growth and development. Additionally, several beta-hexosaminidases, lipoxygenases, and beta-1,3-glucanases were predicted as the main interaction partners of tomato chitinases (Figure 7; Table S8). Beta-hexosaminidase is an important glycosidase, which is involved in important signal transduction, cell division, and cell integrity events [74,75]. Arabidopsis beta-hexosaminidase (AtHEXO1) can generate paucimannosidic N-glycans on vacuolar glycoproteins, while AtHEXO3 functions on secreted glycoproteins [76,77]. They are involved in hydrolysis processes of fruit ripening [78,79]. In this study, three beta-hexosaminidases were predicted to interact with CHI3, CHI9, CHI14, and CHI17, suggesting that the chitinase family proteins may be involved in the molecular function of beta-hexosaminidases in tomato. A similar result has indicated that tomato beta-hexosaminidase can hydrolyze chitin under acidic conditions [79]. Lipoxygenases catalyze the dioxygenation of polyunsaturated fatty acids to form hydroperoxides participating in leaf senescence, synthesis of jasmonic acid, and defense responses to both abiotic and biotic stress [80,81,82]. Solyc01g097270.2 (pi1) was predicted to interact with loxD (Solyc03g122340.2) in this study, implying the coordination or relevance of their functions as described above. As members of the pathogenesis-related protein 2 (PR-2) family, beta-1,3-glucanase catalyzes the hydrolytic cleavage of the beta-1,3-D glucosidic linkages in beta-1,3-glucans. It is well known that plant beta-1,3-glucanase plays important roles in various stress responses as well as developmental processes [83,84]. Interestingly, in our network analysis, one beta-1,3-glucanase protein (LOC543986: Solyc01g008620.2) was observed to interact with both CHI3 and CHI9 (Figure 7). These results indicated the diversity of chitinase binding proteins, which was helpful to understand their functional roles.

## 5. Conclusions

In this study, a comparative analysis of the *chitinase* gene family was performed. Eight groups were divided via phylogenetic analyses. Exon–intron structure and motif distribution were highly conserved in each group, implying their functional conservation. Chromosomal localization indicated that tandem duplication is the main way for plant *chitinases* expansion in evolution. Synteny analyses suggest functional relevance. Selection analyses identified several significant site-specific selective constraints acting on most *chitinase* paralogs after gene duplication, leading to functional divergence. Differential expression patterns of the *chitinase* genes suggested functional divergence, especially for the duplicated genes. Moreover, functional network analyses also identified several potential correlated genes. This study will provide useful insights for further functional investigation of this family gene.

## Figures and Tables

**Table 1:** *Chitinase* genes identified in tomato. Mw/Kda means molecular weight (kilodalton). GRAVY stands for the grand average of hydropathy. S, C, and M mean secretory pathway, chloroplast, and mitochondrion, respectively, in TargetP localization.

Gene ID	Chrome Location	Length	Mw/Kda	pI	GRAVY	Domains	TargetP Localization
Solyc01g095250.1	ch01:86587643..86588539 (+)	298	31.91	4.51	−0.022	GH18	S (0.995)
Solyc01g095260.1	ch01:86590501..86591397 (+)	298	31.95	4.41	−0.019	GH18	S (0.993)
Solyc01g095310.1	ch01:86626834..86627742 (+)	302	32.35	5.34	−0.05	GH18	S (0.994)
Solyc01g097270.2	ch01:88179851..88181214 (−)	212	22.69	8.36	−0.295	GH19	S (0.910)
Solyc01g097280.2	ch01:88183566..88184361 (−)	199	21.49	8.78	−0.37	GH19	S (0.959)
Solyc02g061770.2	ch02:33290113..33291907 (+)	263	28.52	8.69	−0.17	GH19	S (0.953)
Solyc02g082920.2	ch02:46550982..46552140 (+)	253	27.63	5.93	−0.265	GH19	S (0.988)
Solyc02g082930.2	ch02:46554240..46555813 (+)	247	26.58	4.68	−0.228	GH19	S (0.841)
Solyc02g082960.2	ch02:46564405..46566106 (+)	273	30.14	9.56	−0.194	GH19	S (0.887)
Solyc03g116190.1	ch03:65691845..65692213 (−)	122	13.98	8.09	−0.202	GH19	S (0.896)
Solyc03g116230.2	ch03:65723988..65725192 (−)	295	30.59	5.38	−0.196	GH19	S (0.982)
Solyc03g116240.2	ch03:65733982..65736687 (−)	87	9.26	4.94	0.526	GH19	S (0.985)
Solyc04g072000.2	ch04:59066373..59068261 (−)	276	30.01	4.58	−0.294	GH19	S (0.994)
Solyc05g050130.2	ch05:60108919..60110761 (−)	292	31.26	8.46	0.087	GH18	S (0.972)
Solyc06g053380.2	ch06:36128793..36130489 (−)	289	31.81	5.37	−0.284	GH19	S (0.977)
Solyc07g005080.1	ch07:112239..113536 (+)	386	41.83	4.82	−0.125	GH18	Other (0.554); C (0.337)
Solyc07g005090.2	ch07:113980..115328 (−)	371	41.51	8.66	−0.111	GH18	S (0.899)
Solyc07g005100.2	ch07:123941..126335 (+)	376	41.85	9.34	−0.271	GH18	S (0.951)
Solyc07g009500.1	ch07:4618503..4618766 (+)	87	9.49	8.36	−0.298	GH19	S (0.704)
Solyc07g009510.1	ch07:4622506..4622769 (+)	87	9.49	8.36	−0.298	GH19	S (0.704)
Solyc07g009530.1	ch07:4681799..4681990 (+)	63	6.49	7.48	0.733	GH19	S (0.995)
Solyc07g026990.1	ch07:32915139..32915324 (−)	61	7.43	7.77	−0.387	GH19	Other (0.784)
Solyc09g098540.2	ch09:72338607..72340235 (−)	319	35.56	7.95	−0.205	GH19	S (0.964)
Solyc10g017970.1	ch10:6248916..6249098 (−)	60	6.17	5.57	0.497	GH19	S (0.992)
Solyc10g017980.1	ch10:6273805..6273996 (+)	63	6.56	7.48	0.471	GH19	S (0.995)
Solyc10g055790.1	ch10:57408394..57408612 (−)	72	7.72	7.51	0.408	GH19	S (0.976)
Solyc10g055800.1	ch10:57410647..57411787 (−)	329	35.41	8.64	−0.423	GH19	S (0.838)
Solyc10g055810.1	ch10:57417284..57418434 (−)	322	34.35	6.19	−0.248	GH19	S (0.989)
Solyc10g055820.1	ch10:57435941..57437097 (−)	322	34.89	6.36	−0.348	GH19	S (0.975)
Solyc10g068350.1	ch10:65525076..65525336 (−)	86	9.23	7.65	−0.494	GH19	Other (0.600); C (0.419)
Solyc10g074360.1	ch10:57700740..57701259 (−)	154	17.16	8.51	−0.161	GH19	Other (0.677); M (0.523)
Solyc10g074380.1	ch10:57722610..57722870 (−)	64	7.19	9.6	−1.008	GH19	Other (0.504); C (0.363)
Solyc10g074390.1	ch10:57732393..57733097 (−)	216	22.81	8.47	−0.315	GH19	S (0.964)
Solyc10g074400.1	ch10:57738774..57739374 (−)	173	18.43	9.11	−0.381	GH19	S (0.436); M (0.435)
Solyc10g074460.1	ch10:57954087..57956593 (−)	194	20.89	8.36	−0.132	GH19	S (0.966)
Solyc11g005890.1	ch11:710226..711943 (−)	295	33.33	5.7	−0.052	GH18	S (0.876)
Solyc11g020130.1	ch11:10197594..10198487 (+)	163	17.39	7.75	−0.111	GH19	S (0.995)
Solyc11g020450.1	ch11:11116611..11117328 (−)	206	22.18	8.17	−0.675	GH19	M (0.368)
Solyc11g020530.1	ch11:11364445..11364994 (−)	167	18.29	8.47	−0.499	GH19	M (0.470)
Solyc11g072750.1	ch11:55960411..55961439 (−)	342	38.89	6.89	−0.139	GH18	M (0.597)
Solyc11g072760.1	ch11:55962509..55963429 (−)	306	34.63	6.29	−0.037	GH18	S (0.909)
Solyc11g072830.1	ch11:56024533..56025453 (+)	306	33.74	4.79	−0.15	GH18	S (0.974)
Solyc12g098810.1	ch12:66187159..66189951 (+)	328	36.44	6.32	−0.242	GH19	S (0.962)

**Table 2:** Inference of duplication time of *chitinase* paralogous pairs in tomato. *K_a_* and *K_s_* were calculated with K-Estimator 6.0 [49]. The approximate date of the duplication event was inferred from the formula (T = *K_s_*/2λ) as described in methods.

Paralogous Pairs		*K_a_*	*K_s_*	*K_a_*/*K_s_*	Data (Mya)	Duplication Style
*Solyc10g055810.1*	*Solyc10g055820.1*	0.07390	0.35721	0.20688	11.91	Tandem duplication
*Solyc10g074400.1*	*Solyc10g074460.1*	0.07391	0.17341	0.42622	5.78	Tandem duplication
*Solyc02g061770.2*	*Solyc02g082960.2*	0.18678	0.83819	0.22284	27.94	Tandem duplication
*Solyc02g082920.2*	*Solyc02g082930.2*	0.17407	0.63165	0.27579	21.06	Tandem duplication
*Solyc10g055790.1*	*Solyc10g074380.1*	1.35815	0.70977	1.91351	23.66	Tandem duplication
*Solyc10g017970.1*	*Solyc10g017980.1*	0	0.01667	0	0.56	Tandem duplication
*Solyc01g097270.2*	*Solyc01g097280.2*	0.09717	0.57283	0.16963	19.09	Tandem duplication
*Solyc07g009500.1*	*Solyc07g009510.1*	0	0.14145	0	4.72	Tandem duplication
*Solyc11g020450.1*	*Solyc11g020530.1*	0.05676	0.06623	0.85701	2.21	Tandem duplication
*Solyc11g072750.1*	*Solyc11g072760.1*	0.07842	0.2463	0.31839	8.21	Tandem duplication
*Solyc07g005080.1*	*Solyc07g005090.2*	0.46577	1.45233	0.32071	48.41	Tandem duplication
*Solyc01g095250.1*	*Solyc01g095260.1*	0.01671	0.14434	0.11577	4.81	Tandem duplication
*Solyc03g116190.1*	*Solyc07g026990.1*	1.30202	1.25040	1.04128	41.68	Transposition
*Solyc07g009530.1*	*Solyc10g068350.1*	1.10203	0.77691	1.41848	25.89	Transposition
*Solyc04g072000.2*	*Solyc06g053380.2*	0.18915	0.72624	0.26045	24.21	Transposition
*Solyc09g098540.2*	*Solyc12g098810.1*	0.14047	0.67931	0.20678	22.64	Transposition

**Table 3:** Likelihood values and parameter estimates of the *chitinase* genes. Three evolutionary modes—M8 (beta + w ≥ 1), M7 (beta), and M5 (gamma)—were used in this analysis. PSS: positive selection site.

Branches	Models	*K_a_/K_s_*	Log-Likelihood	Number of PSS
GH19-A	M8	0.3927	−15690.8	0
	M7	0.3748	−15691.3	0
	M5	0.4004	−15694.2	22
GH19-B	M8	0.4587	−2023.77	0
	M7	0.5158	−2024.59	0
	M5	0.6367	−2029.54	6
GH19-C	M8	0.3059	−9827.03	0
	M7	0.2989	−9829.36	0
	M5	0.3199	−9831.15	0
GH19-D	M8	0.2091	−5411.06	0
	M7	0.2046	−5414.26	0
	M5	0.2182	−5409.51	0
GH19-E	M8	0.6195	−6088.14	10
	M7	0.4399	−6099.04	0
	M5	0.5484	−6091.2	13
GH18-A	M8	0.5305	−15342.2	47
	M7	0.4669	−15380.5	0
	M5	0.5576	−15367.2	57
GH18-B	M8	0.3478	−5749.57	0
	M7	0.3589	−5751.15	0
	M5	0.3503	−5749.08	0
GH18-C	M8	0.3696	−20704.8	16
	M7	0.3409	−20745.7	0
	M5	0.3923	−20736.3	33

## Supplementary Materials

The following are available online at https://www.mdpi.com/2223-7747/8/3/52/s1, Table S1: Primers using in this study, Table S2: Distribution of the chitinase genes in different species and groups, Table S3: Sequence consensus of 12 motif identified in this study, Table S4: The effects of point mutations on folding stability on the chitinase protein (Solyc10g055820.1), Table S5: The raw transcriptome data (GSE33507) used in this study to investigate the expression profiles of the chitinase genes in different tomato tissues, Table S6: Raw data for the qRT-PCR in this study, Table S7: *cis*-elements analysis of the promoters in the *chitinase* genes. Table S8: Interaction data generated by STRING in this study, Figure S1: Alignment of the tomato chitinase proteins. Chitinase names of different groups in our classification are marked with different colors: GH19-A members in green; GH19-B in saffron yellow; GH19-C in purple; GH19-D in light blue; GH19-E in red; GH18-A in black; GH18-B in dark blue; and GH18-C in deep red, respectively (refer to Figure 2). Chitinase 18 signature is marked with bright green background. Two chitinase 19 signatures are marked with grey and red background. Catalytic residues of chitinase 18 are written in red. Crystallyn signature is marked with yellow background. C-terminal extension for vacuolar targeting is marked with blue background. Pink stands for the catalytic activity of Class I chitinases.
