# Supplementary material for: Comprehensive Analysis of the Chitinase Family Genes in Tomato (Solanum lycopersicum)
Source: Plants (Basel). 2019 Feb 28;8(3):52. doi: 10.3390/plants8030052 (PMC6473868; doi:10.3390/plants8030052)
Supplement: Supplementary file 1 [file plants-08-00052-s001.zip › Supplementary Data/Fig. S1 tomato align.doc]

**Solyc11g005890.1** -------------------------------------MA-K-ILFIFLH------LFALLL----------------------------RI----------RQFCDGKVMMEYIGAT----GKPITFDDV-----------PIDEGIDFHFILSFAIDADS---LGNPQNGKFSPCWAS-

**Solyc11g072830.1** -----------------------------------MELS-K-ILVSFFVIQFLLFHSPIYA-----------------------------A----------APGN-SNIFREYIGAE----FNNVKFSDV-----------PIYSGVEFHFILSFAIDYTMSSSSPSPTNGQFNVFWDSD

**Solyc11g072750.1** MHTTNALLKEKYFNRHLLWLNTNKKYKGSFQLQQIMKSL-K-FCISLLVLQALFNVFPTIE----------------------------AV----------PAFC-PGVFREYIGAE----GKNVTFSDV-----------PINPNIEFHFLLSFAIDYTNT-KSPEPTNGDFLVYWDTD

**Solyc11g072760.1** -----------------------------------MEFF-K-FCISLLVLQTLLTISRITA----------------------------TV----------SPSC-PVVFREYIGAL----GKNVTFSDV-----------PINPNVEFHFLLSFAIDYTNT-ISPEPTNGDFLIYWDTD

**Solyc07g005100.2** -----------------------------------MAN---FIKLFFLFFF-F--------------------------------QQLIFSNGQNNVVKG----------GYWFR--D-----------S-GL----------SL-------------------------N---------

**Solyc07g005080.1** -------------------MMPPTPAPAPF---PLSMP---PIPASIPYPDTP--------------------------------APAPGPAPPLSGIKG----------AYWPSWVA-----------E-KA----------TP-------------------------S---------

**Solyc07g005090.2** -----------------------------------MAN---ILSCCIVFFLAL--------------------------------CSGVMANPHCKGVKG----------AYYPSWAF-----------S-TF----------PP-------------------------S---------

**Solyc05g050130.2** -----------------------------------MSIKY--G-LLYLVLVLLLLSLKLEGG-----------GIVI---YWGQNGNEGTLASTCASNNY-A--IVNIAFLVVFGNGQTPVLNLAGHCNP------------SV-GGCTR------------------LSNDI-------

**Solyc01g095310.1** -----------------------------------MAIKTSFFLHYVPILLILALVARSEAG-----------GIAI---YWGQNGNEGTLAETCATGNY-E--FVNIAFLSTFGNGRNAMLNLAGHCDP------------YTPNGCVN------------------VSSDI-------

**Solyc01g095250.1** -----------------------------------MELQSNRSLALF-SIVILILAASCDAG-----------GIAI---YWGQNGGEGTLAETCATKNY-D--FVNIAFLPTFGNGQQPMIDLSGHCNP------------NV-GECTK------------------LSTDI-------

**Solyc01g095260.1** -----------------------------------MEVQPNRSLALF-SIVILVLAASCDAG-----------GIAI---YWGQNGGEGTLAETCATKNY-D--FVNIAFLPTFGNGQQPMIDLSGHCNP------------NV-GECTK------------------LSTDI-------

**Solyc07g026990.1** ------------------------------------------------------------------------------------------------------------------------------------------------------------------------------------

**Solyc10g074400.1** -------------------MF-----AITL---RMRKTS-KFTTFSLLFSLVLLSAASAQN---------------------------------CGSQGG-GKVCASGQCCSKFGWC----GKTNHHCGS-GNCESQCPGGGPSPV----------------------TGGDL-------

**Solyc10g074460.1** ----------------------------------MRQTS-KFTTFSLLFSLVLLSAASAQN---------------------------------CGSQGG-GKVCASGQCCSKFGWC----GGG------------------PGPGPV--------------------TGGDL-------

**Solyc10g055790.1** --------------------------------------M-RLSACCLLFSLLLLT-VSAEQ---------------------------------CGRQAG-VAHCAAGLCCSKFGWC----GYTDAHCAP-GNCQSQCPPRNYLHLLLLET-----------------------------

**Solyc10g074380.1** ------------------------------------------------------------------------------------------------------------------------------------------------------------------------------------

**Solyc10g074390.1** ----------------------------------MRKTS-KFTTFSLVFSLVLLSAASAQN---------------------------------CGSQGG-GKVCASGQCCSKFGWC----GNTNDHCAS-GNCQSQCPGGGPGPGPD--------------------TGGDL-------

**Solyc10g068350.1** ------------------------------------------------------------------------------------------------------------------------------------------------------------------------------------

**Solyc10g074360.1** -----------------------------------------------------------------------------------------------------------------MAL-----------CTW-KEIF----R--TRPYPN------------------------F-------

**Solyc10g055800.1** --------------------------------------M-RLYAYSLLFSLLLLT-VSAEQ---------------------------------CGRQAG-GARCAAGLCCSNFGWC----GNTNDYCGP-GKCQSQCPSGP-SPKPPTPGPGP--------------SGGDI-------

**Solyc10g055810.1** -----------------------------------MRLS-EFTTLFLLFSVLLLS-ASAEQ---------------------------------CGSQAG-GALCASGLCCSKFGWC----GNTNEYCGP-GNCQSQCPGGP-GP------------------------SGDL-------

**Solyc10g055820.1** -----------------------------------MRHF-EFIVFSLLFSSPLLT-ASAEQ---------------------------------CGVQAG-GALCAAGLCCSKFGWC----GYTDAHCAP-GNCQSQCRDSP-EP------------------------SGDL-------

**Solyc09g098540.2** --------------------------------------M-KMRMIMILFLLVIIG----GF------------------VHGDENESFRKQPTLVKTVKG-TKMCVKDWECNKLS----------------------------------KFC----------------CNLTI-------

**Solyc12g098810.1** -----------------------------------MSAL-KMKVLGFMVIMLSISAIAKGD------------------ENENENESSSKPPILVKMVKG-KKMCVKDWECNVLS----------------------------------RFC----------------CNLTI-------

**Solyc02g061770.2** --------------------------------------------MRLLVLG-----------------------------------------------------------LFSVL----------------------------------CLKCV--------------LSQNI-------

**Solyc02g082960.2** --------------------------------------M-KNK-IMFWGFV-----------------------------------------------------------LFCVL----------------------------------STRGG--------------LTQDV-------

**Solyc02g082920.2** --------------------------------------M-KFNIVSPVALS-----------------------------------------------------------CLFFL----------------------------------FLTGT--------------LAQNA-------

**Solyc02g082930.2** -----------------------------------------------MVLC-----------------------------------------------------------CVFLL----------------------------------FLTGS--------------FAQDV-------

**Solyc03g116240.2** -----------------------------------MK----KTLLALLVLFLLKVVLANEDELSLPFQLPLIGLEA-----------SMGNQVQCGAQN-PGVKCLDGMCCSNCGWC----GNTAAYCSP-GYCLNQSAILPF-------------------------------------

**Solyc03g116230.2** -----------------------------------MKET-AISVLGLLSLFLLEVVSAN--EIING-------TFVLEGINKNASGGVFANGDECGMQANHRSKCPSGMCCSIWGWC----GTTSEYCGS-GFCQNQCTG-PSPHGSCG-MQGG--------------GTK---------

**Solyc11g020130.1** -----------------------------------MKI--NVVALLVLPLFLFMVVSAH--ELSLPFHLPINETFGLVTYSYKG--ECGMHGGRCGWEDG-GRKCPKGVCCIYSGWC----GTTSDYCDV-DKCQSQCSG-PFPQGRCG-WQAD--------------NGS---------

**Solyc11g020450.1** -------------------------------------------------------------------------------MSNTSQCPYPYPQGRCGLEAS-GRKCPKGVCYSYSCWS----GNTLDYYDV-DKCQSQCSG-PFPQGRCE-WQAD--------------NGS---------

**Solyc11g020530.1** -----------------------------------------------------------------------MSYWTVLWYLWFVQCSYPYPQGQCGLEAS-GRKCPKGVCYNYSGLC----GNTLDYYDV-DKCQSQCSG-PFTQGRCG-WQAD--------------NGS---------

**Solyc04g072000.2** -----------------------------------MNFF-SSS--KAFFFLFF----------------------TLSI------ASRLVLAQDCG--------CSSDLCCSKWGYC----GSGNDYCGE--GCQG----GPCF---ST-TPSN--------------------------

**Solyc06g053380.2** -----------------------------------MINF-SSR---ILLILLLTCT------------------IAIII------IPKLASTQYCG--------CTEGLCCSRWGYC----GSGKAYCGQ--GCQG----GPCY---NS-STKN--------------YNHNINN---NN

**Solyc03g116190.1** -------------------------------------MM--MRITIILAFELLVFTMGSD------FDV--RKNETLEG------IDEPYPSGRCGWQAG-GRKCPPKLCCSRHGWC----GTERGCCDP-DFCQSQCSGTPMPPYIAP-FLTA--------------RQQPA-------

**Solyc01g097270.2** -----------------------------------MNKL-SSSTPILLALVLCI------------------------S------LTSVTNAQQCGRQRG-GALCGGNLCCSQFGWC----GSTPEYCSPSQGCQSQCRGG---PTPTP-TPGG--------------G-AQV-------

**Solyc01g097280.2** -----------------------------------MTK------LISINILFVL------------------------I------ISAIANAQQCGRQKG-GALCSGNLCCSQFGWC----GSTPEFCSPSKGCQSRCTGGGSTPTPTP-TPSG--------------SAQNI-------

**Solyc07g009500.1** --------------------------------------M-KFST--TLVFVILA------------------------L------LLTTTYAEQCGRQNG-KRKCPNKLCCSKFGWC----GTSCDYCGS--GCQSNCRKGCATAMFAN-ETVN--------------NNG---------

**Solyc07g009510.1** --------------------------------------M-KFST--TLVFVILA------------------------L------LLTTTYAEQCGRQNG-KRKCPNKLCCSKFGWC----GTSCDYCGS--GCQSNCRKGCATAMFAN-ETVN--------------NNG---------

**Solyc07g009530.1** -----------------------------------MKLM-KFV---VVIVTILA------------------------L------LLSIANAQQCGIQAG-GALCDNGLCCSQFGYC----GTTTAYCGP--GCQSQCN-----------------------------------------

**Solyc10g017970.1** --------------------------------------M-KFF---VLVVTILA------------------------L------LLSVANAQQCGSQAG-GALCANGLCCSQYGYC----GTTPDYCGQ--GCQSQCN-----------------------------------------

**Solyc10g017980.1** -----------------------------------MKMM-KFF---VLVVTILA------------------------L------LLSVANAQQCGSQAG-GALCANGLCCSQYGYC----GTTPDYCGQ--GCQSQCN-----------------------------------------

**Solyc11g005890.1** TLTPNSVASIKSKYPNVKVLASLSGWSIGSKV--------------------------------------------------------------------------LSWYKP-------------IDTQQWISNAFTSLKSI----------------VNTYNLD-----GIDIDYERFP

**Solyc11g072830.1** NLNPSAVSAIKNQHSNVKVALSLGGDSVGGSS---------------------------------------------------------------------------AYFNP-------------SSVDSWVSNAVSSLTNI----------------IKQYNLD-----GIDIDYEHFQ

**Solyc11g072750.1** NLTPSHISSIKAKHKNVKVGMSLGGDTVNGKN---------------------------------------------------------------------------ATFTP-------------TSITSWVSNAIHSITKI----------------VKEYNLD-----AIDIDYEHFN

**Solyc11g072760.1** NLTPSHISSIKAKHKNVRVGMSLGGDTVNGKN---------------------------------------------------------------------------ATFAP-------------TSITSWVRNAIYSITKI----------------AKEYNLD-----AIDIDYEHFN

**Solyc07g005100.2** ----NID---STLFTHLFCAFANL-----NP-----------QSNQL----VISPEDQDSFR--QFTSIVM----------------------RKNPS-------VKTLLSIGGGR---------ANRTAYGIMART-------TNSRKSFIDSSIKLARQLGFH-----GLDLDWE-YP

**Solyc07g005080.1** ----SIP---TAYFTHIFYAFVVP-----DN-----------TSFQL----LISQVDEQTMI--NFTSAIH----------------------SHSPA-------TKAMLSIGG-D---------TGSPVLPSMMSC-------QDNRAAFIKSTIKVSRNYGFD-----GLDLDWE-FP

**Solyc07g005090.2** ----SID---TSLFTHIYYAFLVP-----NN-----------TTFKF----DIDDETSKLLF--NFTSTLR----------------------SS----------VKTLFSVGGGG---------EGPARFSRMAST-------SVSRLSFIKSSIEVARKYKFD-----GFDLDWE-FP

**Solyc05g050130.2** ---RTCQ---SRGIKVML--------SLGG----------GVGSYSLSSADDARNVANYLWN--NYLGGQS----------------------TSRPLGDAILDGID--FD-IERGTTQ----------HWDELA-----RALSGFSQ------------QRKVYLTA--APQCP---FP

**Solyc01g095310.1** ---KSCQ---SGGIKVML--------SIGG----------GAGAYSIASPDDAYQVAAYLWN--NFLGGKS----------------------TSRPLGDAILDGID--FD-LESGEGP----------YWGDLA-----KNLARYNSNR---------GSKKVYLTA--APQCP---FP

**Solyc01g095250.1** ---KSCQ---AKGIKVIL--------SIGG----------GAGSYYLASADDAREVATYLWN--NFLGGQS----------------------TTRPLGDAVLDGID--FD-IEGGTNL----------YWDVLA-----KSLSAYSSM-----------GKKVYLTA--APQCP---FP

**Solyc01g095260.1** ---KSCQ---AKGIKVIL--------SIGG----------GAGSYYLASADDAREVANYLWN--NFLGGQS----------------------TTRPLGDAVLDGID--FD-IEGGTNL----------YWDVLA-----KSLSAYSSM-----------GKKVYLTA--APQCP---FP

**Solyc07g026990.1** ------------------------------------------------------------------------------------------------------------------------------------------------------------------------------------

**Solyc10g074400.1** ----------GSVTSNSMFDQMLK----HRNENSCQ---GKNNFYSY----NPFITASRSFP--GF-GTSG--------------------------DINARKREIAAFFAQTSHETTGGWPSAPDGPNQC---YIITQ-KIR-------------------------------------

**Solyc10g074460.1** ----------GSVISNSMFDQML-----------------------------------KSFP--GF-GTSG--------------------------DINARKREIAAFFALTSHETTGGWPSAPDGPFAWGYCFLRER-GNPGDYCSPS---SQWPCAPGRKYFGRG--PIQISHN-YN

**Solyc10g055790.1** ------------------------------------------------------------------------------------------------------------------------------------------------------------------------------------

**Solyc10g074380.1** ------------------------------------------------------------------------------------------------------------------------------------------------------------------------------------

**Solyc10g074390.1** ----------RSVISNSMFDQML--------------------------------------------------------------------------------------------------------------------------------------------------------NN-YN

**Solyc10g068350.1** ------------------------------------------------------------------------------------------------------------------------------------------------------------------------------------

**Solyc10g074360.1** -----------TCLITFILTFQN--------------------------------------------------------------------------------------------------------------------------------------------------------NN-YK

**Solyc10g055800.1** ----------SSVISNSMFDQMLK----HRNENSCQ---GKNNFYSY----NAFINAARSFR--GF-GTTG--------------------------DNTARKREIAAFFAQTSHETTGGWPSAPDGPYAWGYCFLREQ-GSPGDYCTPS---SQWPCAPGRKYFGRG--PIQISHN-YN

**Solyc10g055810.1** ----------GGVISNSMFDQMLN----HRNDNACQ---GKNNFYSY----NAFVTAAGSFP--GF-GTTG--------------------------DITARKREIAAFLAQTSHETTGGWPTAPDGPYAWGYCFLREQ-GSPGDYCTPS---SQWPCAPGRKYFGRG--PIQISHN-YN

**Solyc10g055820.1** ----------SGVISNSMFDQMLN----HRNDNTCQ---GKNNFYSY----NAFISAAKSFP--GF-GTSG--------------------------DTTARKREIAAFFAQTSHETTGGWPTAPDGPYAWGYCFLREQ-GSPGDYCTPS---SQWPCAPGRKYFGRG--PIQISHN-YN

**Solyc09g098540.2** ----------TDYLDVDQFELLFT----KRNSPVAH---AV-GFWDY----GSFIRAAALYQPLGF-GTTG--------------------------GKKMQMKEIAAFFGHVGSKTSCGYGVATGGPLAYGLCYNKEM-SPSQDYCDEYF-KLTYPCTPGARYYGRG--ALPIYWN-YN

**Solyc12g098810.1** ----------SDFFETYQFENLFS----KRNSPVAH---SA-GFWDY----KSFIIASALFQPLGF-GTTG--------------------------GKFMQMKEIAAFLGHVGSKTSCGYGVATGGPLAWGLCYNKEL-SPSQDYCDDYF-KLTYPCSPGVEYYGRG--ALPIYWN-YN

**Solyc02g061770.2** ----------SSLISKNLFERILV----HRNDAACG---AK-GFYTY----EAFITATKTFA--AF-GTTG--------------------------DTNTRNKEIAAFLAQTSHETTGGWATAPDGPYSWGYCYKQEQ-GSPGDYCASS---QQWPCAPGKKYFGRG--PIQISYN-YN

**Solyc02g082960.2** ----------GALISKDIFERMLL----HRNDANCN---AK-GFYTY----DAFITAARSFA--AF-GTTG--------------------------NTNTRKKEIAAFLAQTSLETTGGWGTAPDGPFSWGYCFKQEQ-GSPPDYCVAN---QQWPCAPGKKYFGRG--PIQISYN-YN

**Solyc02g082920.2** ----------GSIVTRELFEQMLS----FRNNDACP---AK-GFYTY----DAFIAAANSFP--GF-GTAG--------------------------DDTARKKEIAAFFGQTSHETNGGS----AGTFTGGYCFVKQI-EQ------------------SDRYYGRG--PIQLTHQ-SN

**Solyc02g082930.2** ----------GTIVTSDLFNEMLK----NRNDDRCP---AK-GFYTY----DAFIAAANSFP--GF-GTTG--------------------------DDTARKKEIAAFFGQTSHETTGGSL-SADGPFAGGYCFVREG-NQ-----------------MGSGFYGRG--PIQLTGQ-SN

**Solyc03g116240.2** ------------------------------------------------------------------------------------------------------------------------------------------------------------------------------------

**Solyc03g116230.2** -----CP---SG------QCCSLLGWC-GTGSDFCKPEICQSQSDGR----LCPRGQCCSVD--GWCGTTTDYCASGLCQSQCPFTPPPSPPPPSPPPSQYQCGMQNG--GTKCNRTGE-------------CCGISGMCGNTYEYCFPGYC------------------QMQCPGP-YP

**Solyc11g020130.1** -----CP---TG--------------------------------------------VCCSLK--G------------------------------------QCGKQAS--GRKCP-TGV-------------CCSDSGWCGTTSIYCDPNRC------------------QSQCSGP-FP

**Solyc11g020450.1** -----CP---TV--------------------------------------------VC---------GTTSYHCGCGKCQSQCERPYPPSPPS----ITEGPCGKQTG--GRKCP-TGV-------------CCSDSGWCGTTSIYCYPNRC------------------QSQCSGP-FP

**Solyc11g020530.1** -----CP---TV--------------------------------------------VCCSLS--GWCGTTSCHCGCGKCQSQCKRPYPPSPPS----ITEGRCGKQTG--GRKCP-NGV-------------CCSDLGWCGTTSIYYYPNRC------------------QSQCSGP-FP

**Solyc04g072000.2** NNNGVIV---SDVVTNAFFNGIA-----DQGASSCE----GKGFYTR----ERFLEALQSYS--NF-GTVG----------------------ST----DDSKREIAAFFAHVTHETGH-------------MCYINEINGPSGDYCDENNTDYP--CVSGKNYYGRG--PIQLSWN-FN

**Solyc06g053380.2** NNNTNRV---SDIVSESFFNGIA-----NQAASNCE----GKGFYTR----SVFLEALKSYP--EF-ATIG----------------------FS---DDDNKREIAAFFAHVTHETGQ-------------MCYINEINGSSRDYCDMTNSEYP--CVSGQNYYGRG--PIQLSWN-FN

**Solyc03g116190.1** -----RG---MRGIRSFFLNADIVWW-------------------------IY-----YYY-----------------------------------------------------------------------------------------------------------------------

**Solyc01g097270.2** -----RA---T---YHI-YNPQNVGWDLNAVSAYCSTWDANKPYSWR----SK-----YGWT--AFCGPVG----------------------PR---GRDSCGKCL---RVTNTRTGA-------------QTTVRIV-----DQCSNGGLDLDINVFRQIDTDGVGNQQGHLIVN-YQ

**Solyc01g097280.2** -----RA---T---YHI-YNPQNVGWDLNAVSAYCSTWDANKPYSWR----KK-----YGWT--AFCGPVG----------------------PR---GRDSCGKCL---RVTNTRTRA-------------QTTVRIV-----DQCSNGGLDLDVNVFRQIDTDGNGNHQGHLIVN-YQ

**Solyc07g009500.1** --------------EHL-DN---G--DFN-------------------------------------------------------------------------------------------------------------------------------------------------------

**Solyc07g009510.1** --------------EHL-DN---G--DFN-------------------------------------------------------------------------------------------------------------------------------------------------------

**Solyc07g009530.1** ------------------------------------------------------------------------------------------------------------------------------------------------------------------------------------

**Solyc10g017970.1** ------------------------------------------------------------------------------------------------------------------------------------------------------------------------------------

**Solyc10g017980.1** ------------------------------------------------------------------------------------------------------------------------------------------------------------------------------------

**Solyc11g005890.1** KHSESFSHCIGELITL---------L---------KNQSVISIATIAPYYRT----I-----------------------------------LPYIELYNNYGESIDYVNHQFYTDK-----VS-TPRGYLKAFVLRSEQFNKEKVIPSYEVDGRG------------------------

**Solyc11g072830.1** ADPNTFAECIGKLITT---------L---------KNDGVISFASIAPYDDD----E--VQ-------------------------------NHYKTLWKSYSQVIDYVNFQFYAYD-----KGTTVSQFMDYFSTQRSNYEGGRILASFGTDGSG------------------------

**Solyc11g072750.1** ADPDTFAECIGRLLYY---------L---------KQNNVVTYTSIAPYADD----S--VQ-------------------------------VHYLALWRKYGHLIDYVNFQFYAYE-----KGTTILQFLHYFETQMYNYKGGKILVSFGTDNSG------------------------

**Solyc11g072760.1** VDPDTFAECIGRLLYF---------L---------KQNNVISYTSIAPYADD----S--VQ-------------------------------VHYLALWRKYGHLIDYINFQFYAYV-----KGTTIPQFLQYFETQRCNYKGGKILVSFGTNNIG------------------------

**Solyc07g005100.2** ESTIDMTN-LGILLDDFRAAINTEARNSGKASLFL--TSAVSNTPRV--NGL--NYPVQSVARNLDWLNVMSYDFYGPNWSPSQTNSHAQLFDPVSHVSGSDGTASWIQAGVSAQKL------VLGIPFYGYAWQLVNANNHGLRAP-ANGKSNAGSIDDGSMTYNQIKNYIVQNRATTV

**Solyc07g005080.1** IKSEDMLN-LALLLKEWRLAINIESLASSKPALIL--SAAVYFSPNLLLSGI--TYPTEVLSNYLDFLSPMCYNYQG-SWDTSATGAPALLYDKSSNISTSYGISAWKQNGVPSTKL------VMGIPLYGNTWELKDPNDHGIGAP-AVGV---GPGSHGEMSYDDIVTFNSENNASIV

**Solyc07g005090.2** QNKKDMEN-FAILLNEWRVEVKKESLATKRPQLLI--TAAVYFSVDFFLWGEFRSYPVPSINKNLDWINLMFYDYRG-SWDTSATGAQAALFDTKSNVSTSYGLSTWIKAGALRSKL------IMGLPLYGRTWKLKDPNVDGIGAP-AVGV---GPGDEGTLTYREIEKFNEENNAKVV

**Solyc05g050130.2** DSWLNGALSTGLFDYV-----WVQFYNN--PPCQYSAGSAVNLKSYWNKWTA-------IQAGKIFLGLPAAPGAAGSGFIPSDVL--VSQVLPAINGSPKYGGVML---------------------WSKFYD----------N---GY---------------------SSAIKPRV-

**Solyc01g095310.1** DACVGGALNTGVFDYV-----WVQFYNN--PPCQYNQENVTSFQDSWKQWIS----S--IPAKKIFLGLPAAPDAAGSGFIPVADL--TSQLLPTIKGSPKYGGVML---------------------WSKYYD----------DQT-GY---------------------SSSIKTHV-

**Solyc01g095250.1** DAWIGNALKTGLFDYV-----WVQFYNN--PPCQYSSSDISNLEAAWEQWTA----D--IPAKKIFLGLPAAPAAAGSGFIPADDL--TSQVLPSIKNSSKYGGVML---------------------WSKYYD----------DQT-NY---------------------SSSIKSDV-

**Solyc01g095260.1** DAWIGNALKTGLFDYV-----WVQFYNN--PPCQYSSSDFNNLEAAWEQWTA----D--IPATNIFLGLPAAPAAAGSGFIPANDL--ISQVLPSIKNSTKYGGVML---------------------WSKYYD----------DQT-NY---------------------SSSIKSDV-

**Solyc07g026990.1** ------------------------------------------------------------------------------------------------------------------MCHVNDNWVKDRIGYYRMYC----------EIL-GV-----SPGHN--LDCSNQRHFEIAFFHLN-

**Solyc10g074400.1** ------------------------------------------------------------------------------------------------------------------------------------------------------------------------------------

**Solyc10g074460.1** YGPCGRAI-------------GVHIYIY--IYIYIY-EMSTIFFDNSEF-----------------------------------------------------------------------------------------------------------------------------------

**Solyc10g055790.1** ------------------------------------------------------------------------------------------------------------------------------------------------------------------------------------

**Solyc10g074380.1** --------------------------------------------------MT----P--Q-SP-----KPSRHDVIIGRWNPSA-----------------------------ECGRGNDNRVQDRIGFYRRHC----------RIL-GV-----SPGDN--LDCGNQRPFGS-------

**Solyc10g074390.1** YGPCGRAI-------------GVDLLNN--PDLAAT-DPVISFKTAIWFWMT----P--Q-SP-----KPSGHDVIIGRWNPSAG-------DRSANRLPGFGVITNIINGGLECGRGNDNRVQDRIGFYRSYC----------RIL-GV-----SPGDN--LDCGNQRPFGS-------

**Solyc10g068350.1** --------------------------------------------------MN----P--Q-SP-----KPSCHDVIIGRWNPSAG-------DRSANHLPGFGVITNIINGGLECGCGNDNRVQDRIGFYRRYC----------GIL-GV-----STGDN--LDCGNQRSFGS-------

**Solyc10g074360.1** YGPCGRAI-------------GVDLLNN--PDLVAT-DPVISFKTAIWFWMT----P--H-SP-----KSSCHDVIIGRWNPSAG-------DLSANRLPGFGVITNIINGGLECGYGNENRVQDRIGFYRRYC----------GIL-GV-----STGDN--LDCGNQRSFGS-------

**Solyc10g055800.1** YGPCGRAI-------------GADLLNN--PDLVAT-DPVISFKSAIWFWMT----P--Q-SP-----KPSCHDVITGRWQPSGA-------DRAANRLPGFGVITNIINGGLECGRGSDSRVQDRIGFYRRYC----------GIL-GV-----SPGDN--LDCGNQRSFGNGLLVDT-

**Solyc10g055810.1** YGPCGRAI-------------GVDLLNN--PDLVAT-DPVISFKSAIWFWMT----P--Q-SP-----KPSCHDVITGRWQPSGA-------DQAANRVPGFGVITNIINGGLECGHGSDSRVQDRIGFYRRYC----------GIL-GV-----SPGEN--LDCGNQRSFGNGLLVDI-

**Solyc10g055820.1** YGPCGRAI-------------GVDLLNN--PDLVAT-DPSISFKSAIWFWMT----P--Q-SP-----KPSCHDVITGRWQPSNV-------DQAANRLPGFGVITNIINGGLECGHGNDNRVQDRIGFYRRYC----------EIL-GV-----SPGNN--LDCGSQRSFGNGLLVDT-

**Solyc09g098540.2** YGAIGEAL-------------KLNLLDH--PEYIEQ-NATMAFQAAIWRWMN----P--MKKG-----QPSAHDAFVGNWKPTKN-------DTLSKRVPGFGTTMNILYGDGVCGQGDVDAMNNIVSHYLYYL----------DLM-GVGREEAGPHEV--LNCAEQKPFNPTAVAAS-

**Solyc12g098810.1** YGKIGEAL-------------KVDLLNH--PEYIEQ-NATLAFQAAMWQWMN----P--VKKG-----QPSAHDAFVGNWKPTKN-------DTLSKRVPGFGTTMNILYGDNVCNQGDIDSMNNIISHYLYYL----------DLM-GIGREEAGPHEL--LTCAEQKPFNPSSSPAA-

**Solyc02g061770.2** YGAAGSAI-------------GVNLLNN--PDLVAN-DAVVSFKTALWFWMT----A--Q-QP-----KPSAHDVITGRWSPSVA-------DSAAGRVPGFGVITNIINGGMECNRGSNALMDNRIGFYRRYC----------QIL-GV-----DPGNN--LDCANQRPFG--------

**Solyc02g082960.2** YGPAGRAI-------------GVDLLNN--PDAVAN-DAVVSFKTALWFWMT----A--Q-RP-----KPSAHFVITGKWRPTGA-------DLSAGRVSGYGLITNIINGGIECGKGSNPKMENRIGFYKRYL----------ESI-LG-----S---I--WTVLNKRLLASKINYYI-

**Solyc02g082920.2** YERAGQGIG------V-----GQELVNN--PDLVAT-DPIISFKTAIWFWMT----E--Q-DN-----KPSCHNVIIGQWTPSPK-------DTAANRVPGYGVITNIINGQFECGMGPNTAAESRIGFYRRYC----------GML-NV-----PTGEN--LDCNNQKNFAQG------

**Solyc02g082930.2** YDLAGQAI-------------GQDLVNN--PDLVAT-DATVSFKTAIWFWMT----A--Q-GN-----KPSCHDVITGQWTPSAA-------DASANRQPGYGVITNIINGGIECGKGQNPQVEDRIGFYRRYC----------TIL-NV-----APGDN--LDCYDQRNFAEA------

**Solyc03g116240.2** ------------------------------------------------------------------------------------------------------------------------------------------------------------------------------------

**Solyc03g116230.2** EGRCGWQA-------------DGKSCPT--GQCCGN-A--------GW--CG---------------------------------------------IGPGFC---DPIFCQSQCSGAPISTAKRD-GGIRSFL----------LNA-AL-----V------------------------

**Solyc11g020130.1** ------------------------------------------------------------------------------------------------------------------------------------------------------------------------------------

**Solyc11g020450.1** QGRCGWQV-------------YNGPCST--GVCCSL-D--------GW--SG---------------------------------------------TTPAYC---ASGNCQSQCKNTLESTKNRM----KEFF----------------------------------------------

**Solyc11g020530.1** QR----------------------------------------------------------------------------------------------------------------------------------------------------------------------------------

**Solyc04g072000.2** YGPAGQSI-------------GFDGLND--PDIVGR-DGVISFKTALWYWMN----N--C--------------------------------HSLITSGQGFGPTIRAINGQIECDGGNPQTVARRVEYYTQYC----------QQL-GV-----DTGDN--LTC---------------

**Solyc06g053380.2** YGPAGKDI-------------GFDGLND--PDIVSR-DSLISFKTALWYWMN----T--C--------------------------------QPLLSSGQGFGATIRAINGPLECDGALPEPVSRRVQHYVQYC----------KQL-GV-----DTGDN--LTC---------------

**Solyc03g116190.1** ------------------------------------------------------------------------------------------------------------------------------------------------------------------------------------

**Solyc01g097270.2** FVNCGDNV-------------NVPLLS-----VVDR-E----------------------------------------------------------------------------------------------------------------------------------------------

**Solyc01g097280.2** FVDCGDN-----------------------------------------------------------------------------------------------------------------------------------------------------------------------------

**Solyc07g009500.1** ------------------------------------------------------------------------------------------------------------------------------------------------------------------------------------

**Solyc07g009510.1** ------------------------------------------------------------------------------------------------------------------------------------------------------------------------------------

**Solyc07g009530.1** ------------------------------------------------------------------------------------------------------------------------------------------------------------------------------------

**Solyc10g017970.1** ------------------------------------------------------------------------------------------------------------------------------------------------------------------------------------

**Solyc10g017980.1** ------------------------------------------------------------------------------------------------------------------------------------------------------------------------------------

**Solyc11g005890.1** ---------------I--QGDAFFDGLKLLETNGFHVNGAMIYSADASSEDNYIYERKIQAFLLNSTSV-

**Solyc11g072830.1** ---------------GLSPENGFFTACSKLKSKG-DLVGIFVWSADDSKSNGFKYEKQSQGLLAISH---

**Solyc11g072750.1** ---------------GLSPRHGFFDACSILKSRG-TLHGIFIWSADDSIKDHFLYEKLSQNLLASATV--

**Solyc11g072760.1** ---------------GLSPEHGFFDACTILRSQG-KLHGIFIWSADDSMKDHFRYEKLSQNLLASAT---

**Solyc07g005100.2** YNATIVGDYCYSGNTWISYDDTQSVRNKVTYVKGRKLRGYFAWHVAAD-QNWLLSKTASQTWGASPQAVK

**Solyc07g005080.1** YNNETVSTYSYSGTNWIGYDDTNSIRAKIKYAKAQGLGGYFFWALGSD-SNWTLSRTASMAWDHDQS---

**Solyc07g005090.2** YDSATVSAYSVAGTSWIGFDDTNSVAMKLHYAQSQRLRGYFFWAVAGD-KDWKISTTAKQSWIIS-----

**Solyc05g050130.2** ----------------------------------------------------------------------

**Solyc01g095310.1** ----------------------------------------------------------------------

**Solyc01g095250.1** ----------------------------------------------------------------------

**Solyc01g095260.1** ----------------------------------------------------------------------

**Solyc07g026990.1** ----------------------------NSYFEMKKVFRLFP----------------------------

**Solyc10g074400.1** ----------------------------------------------------------------------

**Solyc10g074460.1** ----------------------------------------------------------------------

**Solyc10g055790.1** ----------------------------------------------------------------------

**Solyc10g074380.1** ----------------------------------------------------------------------

**Solyc10g074390.1** ----------------------------------------------------------------------

**Solyc10g068350.1** ----------------------------------------------------------------------

**Solyc10g074360.1** ----------------------------------------------------------------------

**Solyc10g055800.1** ----------------------------M-----------------------------------------

**Solyc10g055810.1** ----------------------------M-----------------------------------------

**Solyc10g055820.1** ----------------------------M-----------------------------------------

**Solyc09g098540.2** ----------------------------------------------------------------------

**Solyc12g098810.1** ----------------------------SS----------------------------------------

**Solyc02g061770.2** ----------------------------------------------------------------------

**Solyc02g082960.2** ----------------------------VK----------------------------------------

**Solyc02g082920.2** ----------------------------------------------------------------------

**Solyc02g082930.2** ----------------------------------------------------------------------

**Solyc03g116240.2** ----------------------------------------------------------------------

**Solyc03g116230.2** ----------------------------------------------------------------------

**Solyc11g020130.1** ----------------------------------------------------------------------

**Solyc11g020450.1** ----------------------------------------------------------------------

**Solyc11g020530.1** ----------------------------------------------------------------------

**Solyc04g072000.2** ----------------------------------------------------------------------

**Solyc06g053380.2** ----------------------------------------------------------------------

**Solyc03g116190.1** ----------------------------------------------------------------------

**Solyc01g097270.2** ----------------------------------------------------------------------

**Solyc01g097280.2** ----------------------------------------------------------------------

**Solyc07g009500.1** ----------------------------------------------------------------------

**Solyc07g009510.1** ----------------------------------------------------------------------

**Solyc07g009530.1** ----------------------------------------------------------------------

**Solyc10g017970.1** ----------------------------------------------------------------------

**Solyc10g017980.1** ----------------------------------------------------------------------

Fig. S1. Alignment of the tomato chitinase proteins. Chitinase names of different groups in our classification are marked with different colors: GH19-A members in green; GH19-B in saffron yellow; GH19-C in purple; GH19-D in light blue; GH19-E in red; GH18-A in black; GH18-B in dark blue; and GH18-C in deepred, respectively (refer to Fig. 2). Chitinase 18 signature is marked with bright green background. Two chitinase 19 signatures are marked with grey and red background. Catalytic residues of chitinase 18 are written in red. Crystallyn signature is marked with yellow background. C-terminal extension for vacuolar targeting is marked with blue background. Pink stands for the catalytic activity of Class I chitinases.
