# Supplementary material for: Comprehensive Analysis of the Chitinase Family Genes in Tomato (Solanum lycopersicum)
Source: Plants (Basel). 2019 Feb 28;8(3):52. doi: 10.3390/plants8030052 (PMC6473868; doi:10.3390/plants8030052)
Supplement: Supplementary file 1 [file plants-08-00052-s001.zip › Supplementary Data/Table S1.doc]

Table S1. Primers using in this study.

| **Genes** | **Primer sequence** (5`) | **Primer sequence** (3`) | **Product length (bp)** | **Ta (**℃) |
| --- | --- | --- | --- | --- |
| *Solyc02g061770.2* | AGCACCAGATGGACCATA | GCTGACTTGAAGCACAGTAA | 83 | 60 |
| *Solyc10g055820.1* | CAAGCAGCTAATCGCCTC | CGATCCTGAACCCTGTTAT | 98 | 60 |
| *Solyc02g082920.2* | TTGGTACTGCTGGTGATGA | ACCACCATTAGTTTCGTGAG | 83 | 60 |
| *Solyc02g082930.2* | GGTGATGATACTGCGCGT | ACAATATCCTCCGGCAAATG | 111 | 60 |
| *Solyc10g017970.1* | CTTTGTGTGCCAATGGGTT | CTGTCCACAGTAATCAGGAGT | 71 | 60 |
| *Solyc12g098810.1* | CCTAGCCAAGATTACTGTGAT | TTCGACTCCGGGAGAGCA | 63 | 60 |
| *Solyc04g072000.2* | CTTCTGGACAAGGATTTGGC | TCAACCCTTCTAGCAACAGT | 94 | 60 |
| *Solyc06g053380.2* | GATTCGATGGGCTTAACGAC | GAGAGGTTGGCAAGTGTTC | 98 | 60 |
| *Solyc01g097270.2* | TCTGGTTCTCTGCATTAGCC | CACAAGTTGCCACCACAT | 90 | 60 |
| *Solyc01g097280.2* | ATATCCGCGATTGCGAAC | AATTGGCTGCAACACAAG | 83 | 60 |
| *Solyc07g005100.2* | GCATCACTATTTCTCACAT | GAGAGTTGGTTTGTGATGGT | 154 | 60 |
| *Actin (Solyc01g104770.2)* | ACATTCAATGTCCCAGCC | TGACACCATCACCAGAATCC | 103 | 60 |

Ta: annealing temperature
