# Supplementary material for: Comprehensive Analysis of the Chitinase Family Genes in Tomato (Solanum lycopersicum)
Source: Plants (Basel). 2019 Feb 28;8(3):52. doi: 10.3390/plants8030052 (PMC6473868; doi:10.3390/plants8030052)
Supplement: Supplementary file 1 [file plants-08-00052-s001.zip › Supplementary Data/Table S2.doc]

Table S2. Distribution of the *chitinase* genes in different species and groups.

|  | Tomato | Arabidopsis | Rice | Total |
| --- | --- | --- | --- | --- |
| GH19-A | 11 | 3 | 11 | 25 |
| GH19-B | 7 | 1 | 0 | 8 |
| GH19-C | 2 | 9 | 4 | 15 |
| GH19-D | 2 | 2 | 3 | 7 |
| GH19-E | 10 | 1 | 1 | 12 |
| GH18-A | 3 | 9 | 2 | 14 |
| GH18-B | 4 | 0 | 3 | 7 |
| GH18-C | 4 | 1 | 27 | 32 |
| Total | 43 | 26 | 51 | **120** |
