# Supplementary material for: Comprehensive Analysis of the Chitinase Family Genes in Tomato (Solanum lycopersicum)
Source: Plants (Basel). 2019 Feb 28;8(3):52. doi: 10.3390/plants8030052 (PMC6473868; doi:10.3390/plants8030052)
Supplement: Supplementary file 1 [file plants-08-00052-s001.zip › Supplementary Data/Table S3.doc]

Table S3: Sequence consensus of 12 motif identified in this study.

|  | **Logo** | **Motif occurrences** | **E-value** | **Sites** | **Width** |
| --- | --- | --- | --- | --- | --- |
| motif 1 |  | SWNYNYGPAGQAIGLDLLNNPDLVATDPVVAFKTALWFWMT | 1.4e-1035 | 42 | 41 |
|  |  |  |  |  |  |
| motif 2 |  | RKREIAAFLAQTSHETTGGWY | 3.3e-503 | 43 | 21 |
|  |  |  |  |  |  |
| motif 3 |  | DVITGRWTPSAADTAAGRVPGFGVITNIINGGLECGHG | 1.2e-575 | 28 | 38 |
|  |  |  |  |  |  |
| motif 4 |  | EGSLREACDTGLYTTVIISFLSVFGH | 3.2e-469 | 32 | 26 |
|  |  |  |  |  |  |
| motif 5 |  | CPNGLCCSQFGWCGTTSDYC | 3.4e-458 | 41 | 20 |
|  |  |  |  |  |  |
| motif 6 |  | DRIGFYRRYCGILGVSPGDNLDCYNQRPF | 5.0e-418 | 24 | 29 |
|  |  |  |  |  |  |
| motif 7 |  | YSLPSSQSAADVADYLWNAFLGGRRAGV | 8.5e-396 | 32 | 28 |
|  |  |  |  |  |  |
| motif 8 |  | AKGFYTYDAFITAANSFPGFG | 1.3e-351 | 42 | 21 |
|  |  |  |  |  |  |
| motif 9 |  | DARLGKALATGLFDRIHVRFYNDPPC | 1.6e-360 | 31 | 26 |
|  |  |  |  |  |  |
| motif 10 |  | WPCAPGKKYYGRGPL | 3.6e-323 | 63 | 15 |
|  |  |  |  |  |  |
| motif 11 |  | RKSFIDSSIKVARSYGFDGLDLAWEYPSNDVEMTNFGKLLEEWRAAVEAE | 1.6e-331 | 14 | 50 |
|  |  |  |  |  |  |
| motif 12 |  | CQSKGVKVLLSIGGG | 1.40E-254 | 46 | 15 |
|  |  |  |  |  |  |
