# Supplementary material for: Comprehensive Analysis of the Chitinase Family Genes in Tomato (Solanum lycopersicum)
Source: Plants (Basel). 2019 Feb 28;8(3):52. doi: 10.3390/plants8030052 (PMC6473868; doi:10.3390/plants8030052)
Supplement: Supplementary file 1 [file plants-08-00052-s001.zip › Supplementary Data/Table S4.doc]

Table S4. The effects of point mutations on folding stability on the chitinase protein (Solyc10g055820.1).

| Position | WT | New | *∆∆*G* | pH | T |
| --- | --- | --- | --- | --- | --- |
| 27 | V | A | -0.24 | 7.0 | 25 |
|  |  | S | -0.68 | 7.0 | 25 |
|  |  | R | -0.98 | 7.0 | 25 |
| 48 | Y | L | -0.29 | 7.0 | 25 |
|  |  | T | -0.71 | 7.0 | 25 |
|  |  | K | -0.47 | 7.0 | 25 |
|  |  | S | -1.10 | 7.0 | 25 |
| 64 | D | G | -0.82 | 7.0 | 25 |
|  |  | V | -0.20 | 7.0 | 25 |
| 67 | E | D | -0.04 | 7.0 | 25 |
|  |  | P | -0.44 | 7.0 | 25 |
|  |  | G | -1.27 | 7.0 | 25 |
|  |  | V | -0.39 | 7.0 | 25 |
| 69 | S | A | -1.50 | 7.0 | 25 |
|  |  | R | -1.14 | 7.0 | 25 |
|  |  | G | -2.57 | 7.0 | 25 |
| 83 | Q | K | -0.66 | 7.0 | 25 |
|  |  | R | -0.22 | 7.0 | 25 |
|  |  | S | -1.04 | 7.0 | 25 |
| 172 | S | T | -1.01 | 7.0 | 25 |
|  |  | A | -0.61 | 7.0 | 25 |
|  |  | P | -0.82 | 7.0 | 25 |
|  |  | Q | -0.90 | 7.0 | 25 |
| 234 | S | G | -1.01 | 7.0 | 25 |
|  |  | Q | -0.89 | 7.0 | 25 |
| 251 | N | A | -1.40 | 7.0 | 25 |
|  |  | P | -0.43 | 7.0 | 25 |
|  |  | G | -1.66 | 7.0 | 25 |
|  |  | V | 1.18 | 7.0 | 25 |
| 254 | Q | L | 0.50 | 7.0 | 25 |
|  |  | S | -0.96 | 7.0 | 25 |
|  |  | T | -1.10 | 7.0 | 25 |
|  |  | A | -0.34 | 7.0 | 25 |
| 277 | H | I | -1.00 | 7.0 | 25 |
|  |  | G | -0.71 | 7.0 | 25 |
|  |  | V | -1.15 | 7.0 | 25 |
|  |  | R | -1.14 | 7.0 | 25 |
| 279 | N | G | -1.99 | 7.0 | 25 |
|  |  | Q | -1.58 | 7.0 | 25 |
|  |  | A | -1.81 | 7.0 | 25 |
|  |  | P | -1.57 | 7.0 | 25 |
|  |  | S | -0.93 | 7.0 | 25 |
| 303 | N | P | -0.74 | 7.0 | 25 |
|  |  | S | -0.66 | 7.0 | 25 |
|  |  | A | -1.81 | 7.0 | 25 |
|  |  | G | -2.79 | 7.0 | 25 |
| 321 | T | S | 0.15 | 7.0 | 25 |
|  |  | L | -1.08 | 7.0 | 25 |

* *∆∆*G: *∆*G(NewProtein)-*∆*G(WildType) in Kcal/mol. *∆∆*G<0: Decrease Stability; *∆∆*G>0: Increase Stability.
