# Supplementary material for: Comprehensive Analysis of the Chitinase Family Genes in Tomato (Solanum lycopersicum)
Source: Plants (Basel). 2019 Feb 28;8(3):52. doi: 10.3390/plants8030052 (PMC6473868; doi:10.3390/plants8030052)
Supplement: Supplementary file 1 [file plants-08-00052-s001.zip › Supplementary Data/Table S6.doc]

Table S6: Raw data for the qRT-PCR in this study.

|  | **Identifier** | **Ct** | **Mean Ct** | **dCt** | **ddCt** | **2(-ddCt)** |
| --- | --- | --- | --- | --- | --- | --- |
|  | Solyc01g104770.2 | 21.53 | 21.6 | / | / | / |
|  |  | 21.17 |  |  |  |  |
|  |  | 22.1 |  |  |  |  |
| CK | Solyc02g061770.2 | 24.43 | 24.67 | 3.07 | 0 | 1 |
|  |  | 25.06 |  |  |  |  |
|  |  | 24.52 |  |  |  |  |
| 4 ℃ | Solyc02g061770.2 | 24.84 | 24.63 | 3.027 | -0.043 | 1.03 |
|  |  | 25.02 |  |  |  |  |
|  |  | 24.03 |  |  |  |  |
| 42 ℃ | Solyc02g061770.2 | 25.37 | 25.51 | 3.906 | 0.836 | 0.56 |
|  |  | 25.94 |  |  |  |  |
|  |  | 25.22 |  |  |  |  |
| Drought | Solyc02g061770.2 | 24.29 | 24.68 | 3.084 | 0.014 | 0.99 |
|  |  | 25.16 |  |  |  |  |
|  |  | 24.59 |  |  |  |  |
| NaCl | Solyc02g061770.2 | 23.88 | 24.08 | 2.48 | -0.59 | 1.51 |
|  |  | 24.17 |  |  |  |  |
|  |  | 24.19 |  |  |  |  |
| Ss-24h | Solyc02g061770.2 | 24.76 | 24.23 | 2.63 | -0.44 | 1.36 |
|  |  | 24.01 |  |  |  |  |
|  |  | 23.92 |  |  |  |  |
| Ss-48h | Solyc02g061770.2 | 23.45 | 23.72 | 2.12 | -0.95 | 1.93 |
|  |  | 23.67 |  |  |  |  |
|  |  | 24.04 |  |  |  |  |
| CK | Solyc10g055820.1 | 21.27 | 21.33 | -0.27 | 0 | 1 |
|  |  | 21.42 |  |  |  |  |
|  |  | 21.3 |  |  |  |  |
| 4 ℃ | Solyc10g055820.1 | 21.57 | 21.45 | -0.15 | 0.12 | 0.92 |
|  |  | 21.12 |  |  |  |  |
|  |  | 21.66 |  |  |  |  |
| 42 ℃ | Solyc10g055820.1 | 23.1 | 23.39 | 1.79 | 2.06 | 0.24 |
|  |  | 24.06 |  |  |  |  |
|  |  | 23.01 |  |  |  |  |
| Drought | Solyc10g055820.1 | 21.28 | 21.13 | -0.472 | -0.202 | 1.15 |
|  |  | 21.42 |  |  |  |  |
|  |  | 20.69 |  |  |  |  |
| NaCl | Solyc10g055820.1 | 20.66 | 20.86 | -0.745 | -0.475 | 1.39 |
|  |  | 21.04 |  |  |  |  |
|  |  | 20.88 |  |  |  |  |
| Ss-24h | Solyc10g055820.1 | 21.41 | 20.99 | -0.61 | -0.34 | 1.27 |
|  |  | 20.19 |  |  |  |  |
|  |  | 21.37 |  |  |  |  |
| Ss-48h | Solyc10g055820.1 | 18.72 | 19.29 | -2.309 | -2.039 | 4.11 |
|  |  | 20.04 |  |  |  |  |
|  |  | 19.11 |  |  |  |  |
| CK | Solyc02g082920.2 | 20.05 | 20.33 | -1.27 | 0 | 1 |
|  |  | 20.18 |  |  |  |  |
|  |  | 20.76 |  |  |  |  |
| 4 ℃ | Solyc02g082920.2 | 20.63 | 20.23 | -1.367 | -0.097 | 1.07 |
|  |  | 19.94 |  |  |  |  |
|  |  | 20.12 |  |  |  |  |
| 42 ℃ | Solyc02g082920.2 | 20.74 | 20.86 | -0.735 | 0.535 | 0.69 |
|  |  | 20.93 |  |  |  |  |
|  |  | 20.91 |  |  |  |  |
| Drought | Solyc02g082920.2 | 19.48 | 19.94 | -1.659 | -0.389 | 1.31 |
|  |  | 20.16 |  |  |  |  |
|  |  | 20.18 |  |  |  |  |
| NaCl | Solyc02g082920.2 | 19.85 | 20.07 | -1.533 | -0.263 | 1.2 |
|  |  | 19.97 |  |  |  |  |
|  |  | 20.39 |  |  |  |  |
| Ss-24h | Solyc02g082920.2 | 19.07 | 19 | -2.60 | -1.33 | 2.51 |
|  |  | 19.34 |  |  |  |  |
|  |  | 18.59 |  |  |  |  |
| Ss-48h | Solyc02g082920.2 | 18.16 | 17.64 | -3.96 | -2.69 | 6.49 |
|  |  | 17.71 |  |  |  |  |
|  |  | 17.05 |  |  |  |  |
| CK | Solyc02g082930.2 | 20.31 | 20.55 | -1.05 | 0 | 1 |
|  |  | 21.07 |  |  |  |  |
|  |  | 20.27 |  |  |  |  |
| 4 ℃ | Solyc02g082930.2 | 20.44 | 20.37 | -1.226 | -0.176 | 1.13 |
|  |  | 20.16 |  |  |  |  |
|  |  | 20.51 |  |  |  |  |
| 42 ℃ | Solyc02g082930.2 | 20.26 | 20.39 | -1.213 | -0.163 | 1.12 |
|  |  | 21.02 |  |  |  |  |
|  |  | 19.89 |  |  |  |  |
| Drought | Solyc02g082930.2 | 20.11 | 20.51 | -1.093 | -0.043 | 1.03 |
|  |  | 20.58 |  |  |  |  |
|  |  | 20.84 |  |  |  |  |
| NaCl | Solyc02g082930.2 | 20.14 | 20.25 | -1.348 | -0.298 | 1.23 |
|  |  | 20.12 |  |  |  |  |
|  |  | 20.49 |  |  |  |  |
| Ss-24h | Solyc02g082930.2 | 23.56 | 23.01 | 1.408 | 2.458 | 0.18 |
|  |  | 22.94 |  |  |  |  |
|  |  | 22.53 |  |  |  |  |
| Ss-48h | Solyc02g082930.2 | 21.2 | 21.18 | -0.42 | 0.63 | 0.65 |
|  |  | 21.03 |  |  |  |  |
|  |  | 21.31 |  |  |  |  |
| CK | Solyc10g017970.1 | 30.13 | 30.62 | 9.02 | 0 | 1 |
|  |  | 31.04 |  |  |  |  |
|  |  | 30.69 |  |  |  |  |
| 4 ℃ | Solyc10g017970.1 | 30.18 | 30.05 | 8.454 | -0.566 | 1.48 |
|  |  | 29.86 |  |  |  |  |
|  |  | 30.11 |  |  |  |  |
| 42 ℃ | Solyc10g017970.1 | 29.11 | 29.78 | 8.188 | -0.832 | 1.78 |
|  |  | 30.04 |  |  |  |  |
|  |  | 30.19 |  |  |  |  |
| Drought | Solyc10g017970.1 | 30.94 | 30.87 | 9.272 | 0.252 | 0.84 |
|  |  | 30.55 |  |  |  |  |
|  |  | 31.12 |  |  |  |  |
| NaCl | Solyc10g017970.1 | 30.06 | 29.76 | 8.164 | -0.856 | 1.81 |
|  |  | 29.44 |  |  |  |  |
|  |  | 29.78 |  |  |  |  |
| Ss-24h | Solyc10g017970.1 | 33.14 | 33.47 | 11.867 | 2.847 | 0.14 |
|  |  | 33.38 |  |  |  |  |
|  |  | 33.89 |  |  |  |  |
| Ss-48h | Solyc10g017970.1 | 29.55 | 29.74 | 8.141 | -0.879 | 1.84 |
|  |  | 29.56 |  |  |  |  |
|  |  | 30.08 |  |  |  |  |
| CK | Solyc12g098810.1 | 26.65 | 27.04 | 5.44 | 0 | 1 |
|  |  | 27.46 |  |  |  |  |
|  |  | 27.01 |  |  |  |  |
| 4 ℃ | Solyc12g098810.1 | 26.9 | 26.83 | 5.226 | -0.214 | 1.16 |
|  |  | 27.11 |  |  |  |  |
|  |  | 26.68 |  |  |  |  |
| 42 ℃ | Solyc12g098810.1 | 26.43 | 26.38 | 4.781 | -0.659 | 1.58 |
|  |  | 26.22 |  |  |  |  |
|  |  | 26.49 |  |  |  |  |
| Drought | Solyc12g098810.1 | 27.62 | 27.21 | 5.608 | 0.168 | 0.89 |
|  |  | 27.37 |  |  |  |  |
|  |  | 26.64 |  |  |  |  |
| NaCl | Solyc12g098810.1 | 27.06 | 27.03 | 5.426 | -0.014 | 1.01 |
|  |  | 27.15 |  |  |  |  |
|  |  | 26.88 |  |  |  |  |
| Ss-24h | Solyc12g098810.1 | 29.32 | 29.56 | 7.963 | 2.523 | 0.17 |
|  |  | 30.14 |  |  |  |  |
|  |  | 29.22 |  |  |  |  |
| Ss-48h | Solyc12g098810.1 | 28.26 | 28.62 | 7.022 | 1.582 | 0.34 |
|  |  | 28.47 |  |  |  |  |
|  |  | 29.13 |  |  |  |  |
| CK | Solyc04g072000.2 | 20.45 | 20.39 | -1.21 | 0 | 1 |
|  |  | 20.75 |  |  |  |  |
|  |  | 19.97 |  |  |  |  |
| 4 ℃ | Solyc04g072000.2 | 21.11 | 20.64 | -0.958 | 0.252 | 0.84 |
|  |  | 20.73 |  |  |  |  |
|  |  | 20.08 |  |  |  |  |
| 42 ℃ | Solyc04g072000.2 | 20.95 | 20.82 | -0.776 | 0.434 | 0.74 |
|  |  | 20.84 |  |  |  |  |
|  |  | 20.67 |  |  |  |  |
| Drought | Solyc04g072000.2 | 22.11 | 22.08 | 0.479 | 1.689 | 0.31 |
|  |  | 22.04 |  |  |  |  |
|  |  | 22.09 |  |  |  |  |
| NaCl | Solyc04g072000.2 | 19.46 | 19.84 | -1.756 | -0.546 | 1.46 |
|  |  | 20.15 |  |  |  |  |
|  |  | 19.91 |  |  |  |  |
| Ss-24h | Solyc04g072000.2 | 20.49 | 20.38 | -1.22 | -0.01 | 1.01 |
|  |  | 20.28 |  |  |  |  |
|  |  | 20.37 |  |  |  |  |
| Ss-48h | Solyc04g072000.2 | 19.27 | 19.11 | -2.489 | -1.279 | 2.43 |
|  |  | 19.34 |  |  |  |  |
|  |  | 18.72 |  |  |  |  |
| CK | Solyc06g053380.2 | 30.44 | 30.24 | 8.64 | 0 | 1 |
|  |  | 30.18 |  |  |  |  |
|  |  | 30.1 |  |  |  |  |
| 4 ℃ | Solyc06g053380.2 | 29.85 | 30.12 | 8.516 | -0.124 | 1.09 |
|  |  | 30.06 |  |  |  |  |
|  |  | 30.45 |  |  |  |  |
| 42 ℃ | Solyc06g053380.2 | 30.64 | 30.09 | 8.489 | -0.151 | 1.11 |
|  |  | 29.37 |  |  |  |  |
|  |  | 30.26 |  |  |  |  |
| Drought | Solyc06g053380.2 | 30.7 | 30.09 | 8.489 | -0.151 | 1.11 |
|  |  | 29.41 |  |  |  |  |
|  |  | 30.16 |  |  |  |  |
| NaCl | Solyc06g053380.2 | 30.68 | 30.17 | 8.57 | -0.07 | 1.05 |
|  |  | 29.48 |  |  |  |  |
|  |  | 30.35 |  |  |  |  |
| Ss-24h | Solyc06g053380.2 | 31.6 | 31.7 | 10.102 | 1.462 | 0.36 |
|  |  | 31.66 |  |  |  |  |
|  |  | 31.84 |  |  |  |  |
| Ss-48h | Solyc06g053380.2 | 30.61 | 30.68 | 9.08 | 0.44 | 0.74 |
|  |  | 30.55 |  |  |  |  |
|  |  | 30.88 |  |  |  |  |
| CK | Solyc01g097270.2 | 17.43 | 17.71 | -3.89 | 0 | 1 |
|  |  | 18.01 |  |  |  |  |
|  |  | 17.69 |  |  |  |  |
| 4 ℃ | Solyc01g097270.2 | 17.73 | 17.72 | -3.876 | 0.014 | 0.99 |
|  |  | 18.04 |  |  |  |  |
|  |  | 17.39 |  |  |  |  |
| 42 ℃ | Solyc01g097270.2 | 17.62 | 17.75 | -3.846 | 0.044 | 0.97 |
|  |  | 17.79 |  |  |  |  |
|  |  | 17.84 |  |  |  |  |
| Drought | Solyc01g097270.2 | 17.73 | 17.78 | -3.816 | 0.074 | 0.95 |
|  |  | 17.96 |  |  |  |  |
|  |  | 17.65 |  |  |  |  |
| NaCl | Solyc01g097270.2 | 17.45 | 17.63 | -3.974 | -0.084 | 1.06 |
|  |  | 17.96 |  |  |  |  |
|  |  | 17.48 |  |  |  |  |
| Ss-24h | Solyc01g097270.2 | 17.23 | 17.16 | -4.439 | -0.549 | 1.46 |
|  |  | 17.2 |  |  |  |  |
|  |  | 17.05 |  |  |  |  |
| Ss-48h | Solyc01g097270.2 | 16.48 | 16.4 | -5.199 | -1.309 | 2.48 |
|  |  | 16.11 |  |  |  |  |
|  |  | 16.61 |  |  |  |  |
| CK | Solyc01g097280.2 | 25.87 | 25.94 | 4.34 | 0 | 1 |
|  |  | 26.16 |  |  |  |  |
|  |  | 25.79 |  |  |  |  |
| 4 ℃ | Solyc01g097280.2 | 26.21 | 26.08 | 4.476 | 0.136 | 0.91 |
|  |  | 25.89 |  |  |  |  |
|  |  | 26.14 |  |  |  |  |
| 42 ℃ | Solyc01g097280.2 | 25.77 | 25.86 | 4.256 | -0.084 | 1.06 |
|  |  | 25.93 |  |  |  |  |
|  |  | 25.88 |  |  |  |  |
| Drought | Solyc01g097280.2 | 26.19 | 26.32 | 4.717 | 0.377 | 0.77 |
|  |  | 26.28 |  |  |  |  |
|  |  | 26.49 |  |  |  |  |
| NaCl | Solyc01g097280.2 | 26.22 | 26.06 | 4.46 | 0.12 | 0.92 |
|  |  | 26.01 |  |  |  |  |
|  |  | 25.95 |  |  |  |  |
| Ss-24h | Solyc01g097280.2 | 28.87 | 28.89 | 7.295 | 2.955 | 0.13 |
|  |  | 29.06 |  |  |  |  |
|  |  | 28.74 |  |  |  |  |
| Ss-48h | Solyc01g097280.2 | 26.7 | 26.72 | 5.121 | 0.781 | 0.58 |
|  |  | 26.65 |  |  |  |  |
|  |  | 26.81 |  |  |  |  |
| CK | Solyc07g005100.2 | 33.58 | 32.89 | 11.29 | 0 | 1 |
|  |  | 32.73 |  |  |  |  |
|  |  | 32.36 |  |  |  |  |
| 4 ℃ | Solyc07g005100.2 | 32.83 | 32.81 | 11.206 | -0.084 | 1.06 |
|  |  | 33.11 |  |  |  |  |
|  |  | 32.49 |  |  |  |  |
| 42 ℃ | Solyc07g005100.2 | 33.52 | 33.45 | 11.846 | 0.556 | 0.68 |
|  |  | 33.21 |  |  |  |  |
|  |  | 33.62 |  |  |  |  |
| Drought | Solyc07g005100.2 | 32.29 | 32.35 | 10.754 | -0.536 | 1.45 |
|  |  | 32.33 |  |  |  |  |
|  |  | 32.43 |  |  |  |  |
| NaCl | Solyc07g005100.2 | 32.46 | 32.53 | 10.934 | -0.356 | 1.28 |
|  |  | 32.42 |  |  |  |  |
|  |  | 32.71 |  |  |  |  |
| Ss-24h | Solyc07g005100.2 | 32.76 | 32.9 | 11.3 | 0.01 | 0.99 |
|  |  | 33.05 |  |  |  |  |
|  |  | 32.89 |  |  |  |  |
| Ss-48h | Solyc07g005100.2 | 31.56 | 31.53 | 9.93 | -1.36 | 2.57 |
|  |  | 31.77 |  |  |  |  |
|  |  | 31.26 |  |  |  |  |
